# Spatial distribution of spinal cord fMRI activity with electrocutaneous stimulation

**DOI:** 10.1162/IMAG.a.1383

**Published:** 2026-10-01

**Authors:** Sandrine Bédard, Merve Kaptan, Teresa Indriolo, Christine S.W. Law, Dario Pfyffer, Lindsay Lee, John Ratliff, Serena Hu, Suzanne Tharin, Zachary A. Smith, Gary H. Glover, Sean Mackey, Julien Cohen-Adad, Kenneth A. Weber II

**Affiliations:** NeuroPoly Lab, Institute of Biomedical Engineering, Polytechnique Montreal, Montreal, Quebec, Canada; Division of Pain Medicine, Department of Anesthesiology, Perioperative and Pain Medicine, Stanford School of Medicine, Palo Alto, CA, United States; Department of Neurosurgery, Stanford University School of Medicine, Palo Alto, CA, United States; Department of Orthopaedic Surgery, Stanford University School of Medicine, Palo Alto, CA, United States; Department of Neurosurgery, University of Oklahoma Health Sciences Center, Oklahoma City, OK, United States; Department of Radiology, Stanford University School of Medicine, Palo Alto, CA, United States; Mila—Quebec AI Institute, Montreal, Quebec, Canada; Research Center, Ste-Justine Hospital University Centre, Montreal, Quebec, Canada; Functional Neuroimaging Unit, CRIUGM, University of Montreal, Montreal, Quebec, Canada

**Keywords:** spinal cord, fMRI, dermatomes, electrocutaneous stimulation, spatial normalization

## Abstract

Sensory organization at the spinal segment level is commonly inferred from dermatomal maps that assume a fixed correspondence between cutaneous regions and spinal segments. However, based on the complexities of spinal neuroanatomy and neurophysiology, the distribution of sensory signals within the cord may be broader and less segment-specific than dermatomal maps suggest, leaving the segment-level localization of sensory-evoked activity in humans uncertain. Spinal cord functional magnetic resonance imaging (fMRI) is currently the only technique capable of noninvasively mapping sensory activity with high spatial resolution in the human spinal cord. However, its application remains technically challenging and is limited by the uncertainty in segmental localization. In this study, we leveraged recent advancements in spinal cord fMRI, including spinal nerve rootlet–based spatial normalization, to investigate how sensory information is represented and distributed within the human spinal cord during electrocutaneous stimulation of the third digit of the right hand (i.e., C7 dermatome). Forty healthy adults were scanned with electrocutaneous stimulation at four individualized intensities across multiple runs to quantify (i) the rostrocaudal distribution of sensory-evoked activity, (ii) intensity-dependent changes in detectability and localization, and (iii) the effect of normalization strategy on segmental localization. Across participants, stimulation produced activation localized in the lower cervical cord (e.g., C6–C8), with the most consistent segmental localization near C7. Stronger stimulation increased detectability and produced more consistent segmental localization across participants. Importantly, normalization that incorporated nerve rootlet landmarks sharpened localization and improved sensitivity relative to conventional intervertebral disc–based alignment. This highlights the value of functionally relevant anatomical landmarks for group inference in the spinal cord. Responses were strongest in the initial run and attenuated with repetition, suggesting habituation or adaptation that can bias multi-run paradigms if unmodeled. Together, our results define practical acquisition and analysis conditions (e.g., stimulation strength, anatomical alignment strategy, and run structure) under which segment-level spinal sensory responses can be detected, thereby supporting more reliable studies of human spinal cord future basic and translational studies, including pain mechanisms, sensory function, and spinal injury.

## 1 Introduction

Sensory organization at the spinal segment level is commonly inferred using dermatomal maps that assume a fixed correspondence between cutaneous regions and spinal segments. Conventional dermatomal maps, largely derived from manual drawings, vary considerably and fail to account for inter-individual variability (Lee et al., 2008). Moreover, there remains ambiguity regarding what dermatomes actually represent anatomically—spinal nerves, spinal cord segments, dorsal root ganglia, or dorsal horns (Lee et al., 2008).

Modern spinal anatomy and physiology suggest that sensory-evoked activity may be broader and less segment-specific than dermatomal maps imply. Afferents entering a given dorsal root can arise from multiple peripheral nerves, and single nerves can contribute to multiple roots; moreover, intersegmental anastomoses between cervical dorsal roots (present in ~61% of individuals) can shift entry to adjacent segments (Lissarrague et al., 2025; Moriishi et al., 1989). After entry, primary afferents may ascend or descend several segments within Lissauer’s tract before terminating in the dorsal horn, further widening the rostrocaudal spread of input (Coggeshall et al., 1981; Light & Perl, 1979; Sugiura et al., 1986). Within the spinal cord, sensory signals are transformed by interneuronal spinal networks (Chung & Coggeshall, 1983; Nathan & Smith, 1959; Pierrot-Deseilligny & Marchand-Pauvert, 2002; Soteropoulos et al., 2013; Willis & Westlund, 1997) and descending supraspinal pathways (Stenner et al., 2025) that relay, integrate, and modulate input within and between spinal segments. The spatial location of sensory activity may also depend on the stimulus intensity. In the spinal cord, it remains unclear whether intensity is reflected primarily by increased firing rates, recruitment of additional afferent fibers, engagement of broader interneuronal and supraspinal pathways, or some combination of these mechanisms, particularly across the transition from innocuous to noxious sensation (Summers et al., 2010).

Together, these considerations raise fundamental questions regarding the spinal cord’s role in sensory processing and the validity and reliability of dermatomal maps used clinically: How spatially localized are sensory representations in the human spinal cord, how does their extent change with stimulus intensity, and how much of the observed pattern reflects segmental organization versus distributed spinal processing? Addressing these questions requires methods capable of measuring spinal cord activity in humans with sufficient spatial specificity.

Spinal cord functional magnetic resonance imaging (fMRI) is the only technique to noninvasively map sensory activity with high spatial resolution in the human spinal cord. Using blood-oxygen-level–dependent (BOLD) signals as a proxy for neural activity, spinal cord fMRI can be used to localize and quantify the spatial extent of spinal activity, but remains technically challenging (Kinany et al., 2023). A persistent methodological challenge has been spatial normalization, which is a method used to align anatomy across individuals and an essential step for group-level analyses. Spinal cord fMRI studies have relied on spine anatomy (i.e., vertebral or intervertebral disc levels) for spatial normalization. This approach is inherently inaccurate because of varying correspondence between vertebral and spinal cord segment levels across individuals due to differences in spinal cord length relative to the vertebral column (Cadotte et al., 2015). To overcome this challenge, we recently introduced a spinal nerve rootlet–based spatial normalization method, designed to improve the alignment across individuals by directly targeting neuroanatomical landmarks relevant to segmental organization. This approach improved group-level localization of motor task-related fMRI activity but has not yet been evaluated using a spatially localized sensory stimulus (Bédard, Valošek, et al., 2025).

In this study, we leverage recent advancements in spinal cord fMRI including rootlet-based spatial normalization to investigate how sensory information is represented and distributed within the human spinal cord during electrocutaneous sensory stimulation of the third digit of the right hand (i.e., C7 dermatome) at multiple individualized intensities across multiple stimulation runs. We first evaluate the impact of anatomical alignment based on spinal segment levels. We then characterize intensity-dependent encoding, and finally, we assess response reliability and the presence of spinal habituation/adaptation or sensitization. Together, these experiments test how anatomical alignment, stimulus intensity, and repeated exposures shape the apparent segmental organization, spatial extent, and reliability of sensory-evoked spinal activity, providing new insight into human sensory processing in the spinal cord. These results also define practical acquisition and analysis conditions for more reliable segment-level mapping of human spinal sensory processing.

## 2 Materials and Methods

### 2.1 Participants

Forty healthy adult volunteers were recruited (age = 38.8 ± 14.5, 50% female). Exclusion criteria included contraindications to MRI, pain conditions, opioid use, previous spine surgery, ongoing spine conditions or known peripheral nerve compression, major physical trauma to the spine, cancer, and major medical, psychiatric, and neurological conditions. Inclusion criteria included an age between 20 and 79 years old, right-handedness, and fluency in English. Eligible volunteers were informed about the study protocol and provided written informed consent before participation. All participants received compensation, and the study was approved by the Stanford University Institutional Review Board.

### 2.2 Image acquisition

All imaging was performed in a supine position on a 3T GE SIGNA Premier scanner equipped with a custom 56-channel head-neck coil (designed and built by NeuroPoly Lab, Polytechnique Montreal) at the Richard M. Lucas Center for Imaging at Stanford University. A SatPad cervical collar was also used to improve B0 field homogeneity (De Leener et al., 2020). Physiological measurements were collected using a pulse oximeter placed on the left index finger and a respiratory belt positioned around the abdomen. Spinal cord fMRI BOLD data were acquired using a 2D (axial) spatially selective reduced field-of-view (FOV) pulse GE-EPI sequence with dynamic per-slice shimming (21 slices, 1.25 mm × 1.25 mm × 4.00 mm, TR = 1.7 seconds, bandwidth = 250 kHz, TE = 30 ms) (Islam et al., 2019). The pulse sequence was designed and implemented under the KSFoundation.^1^ The FOV was centered at the C5–C6 intervertebral disc and oriented obliquely to remain perpendicular to the spinal cord, spanning approximately from C5 to T1 spinal segment levels.

For shimming, both axial (FOV = 22 cm, matrix size = 128 × 128, ΔTE = 1 ms) and sagittal (FOV = 30 cm, matrix size = 256 × 64, ΔTE = 1 ms, one slice, during a 3 seconds breath-hold) B0 field maps were acquired to calculate slicewise shims using the methods proposed by Islam et al. (2019). A high-resolution sagittal T2-weighted (T2w) structural scan was acquired for spatial normalization using a 3D turbo spin-echo sequence (CUBE) with the following parameters: TR = 2.5 seconds, TE = 128 ms, matrix size: 64 × 320 × 320, resolution: 0.62 mm × 0.62 mm × 0.62 mm, and bandwidth = 520.8 kHz (Cohen-Adad et al., 2021). This image enables clear visualization of the spinal rootlets (Valošek et al., 2024) and intervertebral discs.

### 2.3 Functional MRI experiment

An overview of the experimental setup is provided in Figure 1. Electrocutaneous stimulations were applied to the third digit of the right hand (C7 dermatome) using MRI-compatible pre-gelled Ag/AgCl ring electrodes with 50 cm lead wires (Cadwell Industries, Kennewick, WA, USA) and a BIOPAC System located outside the scanner room. Four individualized stimulation intensities, evenly spaced between each participant’s sensory threshold and maximum tolerable intensity, were used for the fMRI experiment. Thresholds were determined in a single ascending series (0.2–10 mA, 0.2 mA steps) using a 2-second on / 2-second off pattern. Participants squeezed a hand-held ball at first sensation (sensory threshold) and again when they reached the maximum level of electrical stimulation they judged they could tolerate, at which point the stimulation was stopped and the intensity recorded (maximum tolerable intensity). Pain intensity was assessed using a verbal analog scale (0–10; 0 = no pain, 10 = worst pain imaginable). Pain ratings at both thresholds were reported at the end of the thresholding procedure, and additional pain ratings were reported at the end of each functional MRI run. Subsequent stimulation levels were selected between 0.2 mA above detection and 0.2 mA below tolerance. Thresholding was performed in the MRI scanner at the beginning of the imaging session. As the objective was to identify an individualized stimulation range extending from sensory threshold to the participant’s maximum tolerable intensity, rather than to determine sensory or pain thresholds, a single ascending series was used to avoid delivering stimuli above tolerable levels.

**Fig. 1.**
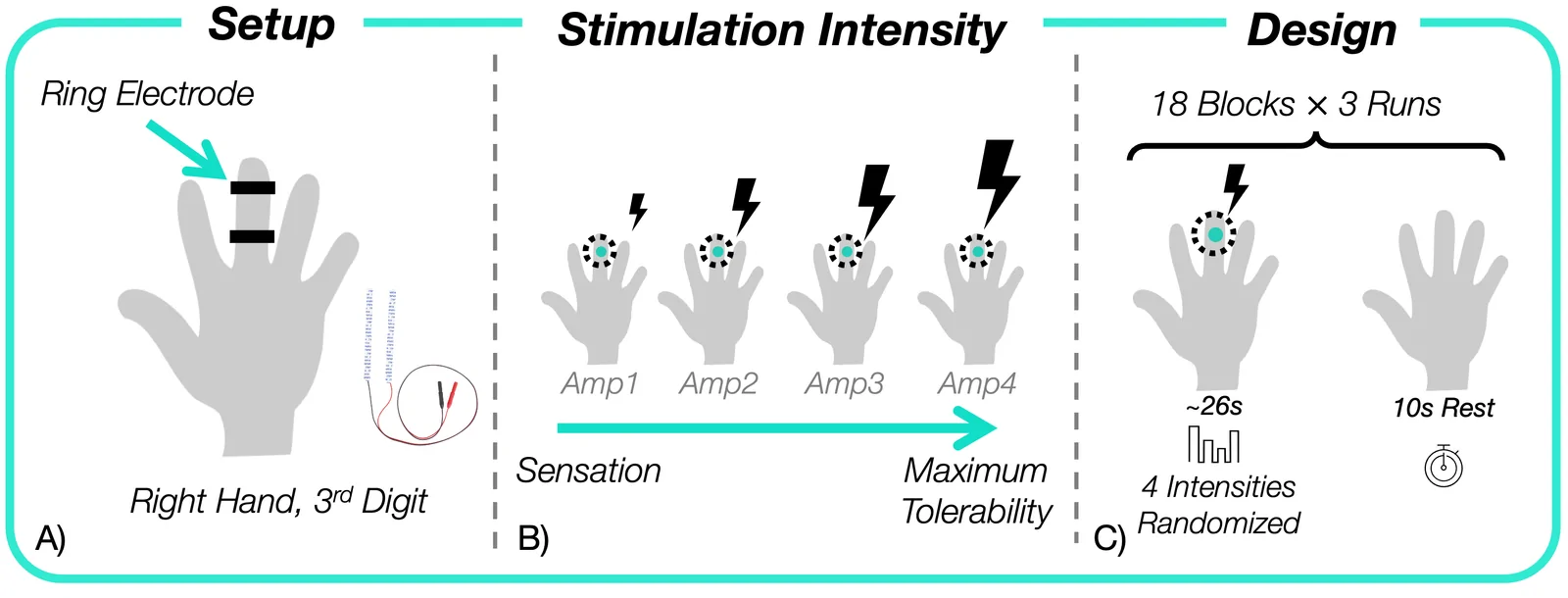
Experimental setup. (A) MRI-compatible Biopac system and Ag/AgCl ring electrodes on the third digit of the right hand. (B) Four individually determined electrocutaneous stimulation intensities between sensory threshold and maximum tolerable intensity. (C) Each run (~10 minutes, 376 volumes) included 18 blocks, with 10 seconds rest periods. Each block delivered four randomized monophasic pulses (100 Hz, pulse width = 0.2 ms, duration = 5 seconds).

Each run consisted of 18 stimulation blocks, each separated by a 10-second rest period. Within each block, four stimulations were delivered using monophasic pulses (100 Hz, pulse width = 0.2 ms, duration = 5 seconds), with the order of the intensity levels randomized within the block and interleaved by 2-second intervals. Participants completed three runs, each lasting 10 minutes, for a total of 376 fMRI volumes. After each run, participants reported perceived pain intensity using the same verbal 0–10 scale described above to be used as a secondary outcome measure.

### 2.4 Data processing

#### 2.4.1 Preprocessing

An overview of the processing pipeline is presented in Figure 2 and available on Github: https://github.com/kennethaweberii/Dermatomal_Mapping_R01/releases/tag/r20260213. The pipeline includes motion correction, physiological noise removal, slice-timing correction, temporal filtering, and spatial normalization. The same preprocessing was applied to all three runs and detailed below.

**Fig. 2.**
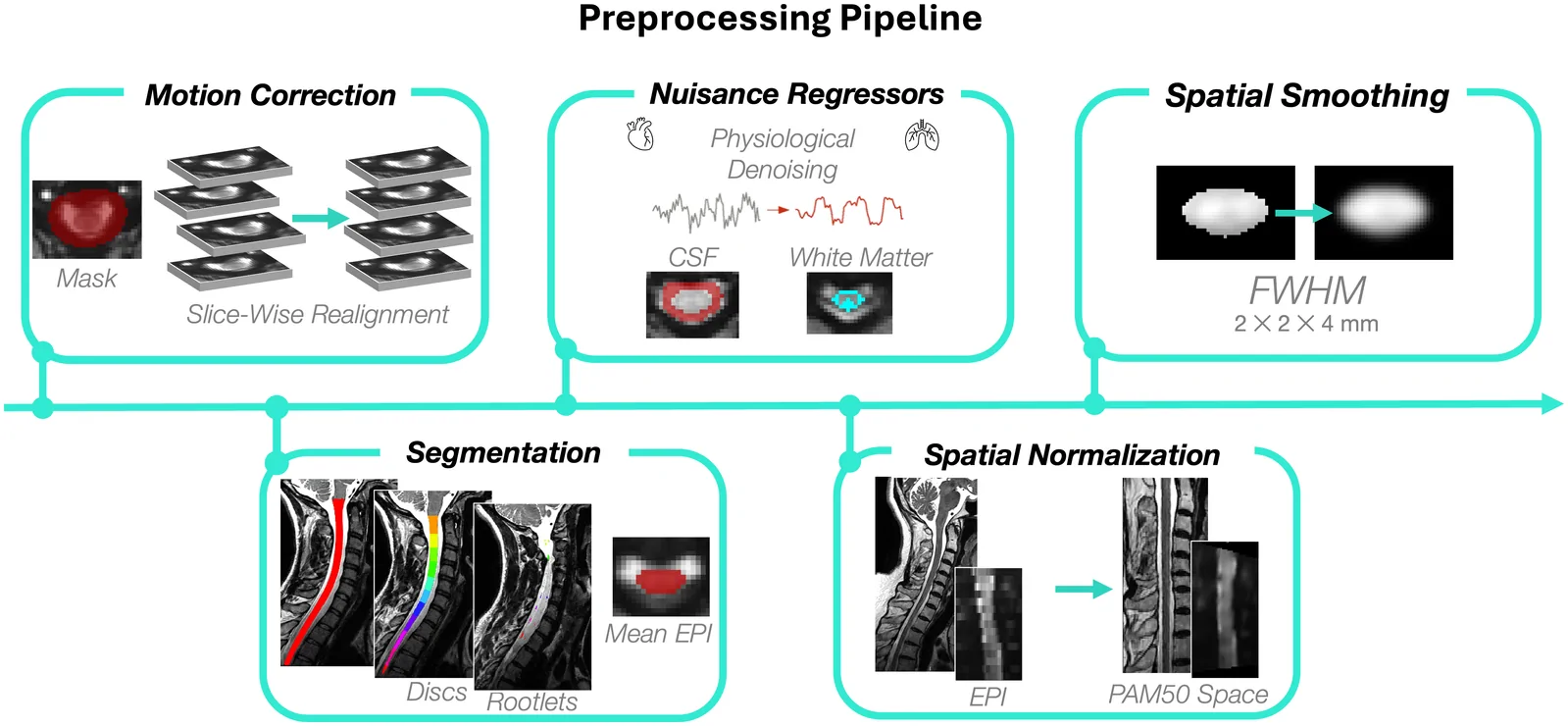
Processing pipeline for fMRI of the spinal cord. Slicewise motion correction of the EPI time series was applied within a mask surrounding the spinal cord. The spinal cord, nerve rootlets, and intervertebral discs were segmented/labeled from the T2w image and the spinal cord was segmented from the mean motion corrected EPI image. Physiological noise, cerebrospinal fluid, and white matter signals were removed. The T2w image was registered to the PAM50 template based on rootlets and discs, and spatial smoothing was applied to the EPI image in the PAM50 template space.

Image processing was performed using the Spinal Cord Toolbox (SCT) v7.0 (De Leener et al., 2017) and FSL v6.0 (Jenkinson et al., 2012). The functional spinal cord time series was first motion corrected using FSL’s FLIRT tool (Jenkinson et al., 2012) in two steps. A mask around the spinal canal was created by dilating the automatic segmentation of the spinal cord obtained using sct_deepseg sc_epi (Banerjee et al., 2025), to restrict the analysis to the spinal column. First, each axial slice of the functional time series was realigned to the mid-volume using 2D rigid-body registration. A mean image was computed, and all volumes were realigned to this mean image in a second round of motion correction (Weber et al., 2014). Given the interleaved 2D acquisition, motion correction was performed slice-wise without an initial 3D rigid-body registration to avoid through-slice interpolation. Each step was visually inspected to ensure accurate alignment across volumes using sct_qc. Motion outliers were identified using FSL’s fsl_motion_outliers and included as confound regressors in the first-level statistical analysis later. Motion outliers were measured using DVARS, which corresponds to the root mean square of the temporal changes in the fMRI voxelwise signal at each time point, with a threshold defined as the 75th percentile plus 1.5 times the interquartile range. Mean temporal signal-to-noise ratio (tSNR) maps were computed following motion correction and compared across runs using a Bonferroni-corrected paired t-test.

The spinal cord was segmented using sct_deepseg sc_epi on the motion-corrected mean functional image (Banerjee et al., 2025). The segmentation was visually assessed using sct_qc (Valošek & Cohen-Adad, 2024) and manually corrected in cases of major errors (e.g., under or over-segmentation) with FSLeyes v1.12.4 (Jenkinson et al., 2012). A spinal canal mask was derived using sct_propseg (De Leener et al., 2014). The cerebrospinal fluid (CSF) mask was computed by subtracting the corrected spinal cord mask from the canal mask.

Spatial normalization relies on the high-resolution T2w structural image. The spinal cord and cervical spinal rootlets (dorsal and ventral, from C2-T1 spinal segment levels) were automatically segmented using sct_deepseg spinalcord (Bédard, Karthik, et al., 2025; Karthik et al., 2025), and sct_deepseg rootlets_t2, respectively (Krejci et al., 2025; Valošek et al., 2024). Vertebral levels were also automatically labeled using sct_label_vertebrae to allow further comparison of disc-based vs. rootlet-based normalization in fMRI analysis (Bédard, Valošek, et al., 2025). The spinal cord was registered to the PAM50 template using both disc- and rootlets-based approaches (sct_register_to_template -lrootlet) (Bédard, Valošek, et al., 2025; De Leener et al., 2018). Figure 3 shows the correspondence between spinal and vertebral segments along with the registration pipeline. The resulting warping field served as an initial transform to align the PAM50 template to the mean functional image on a slicewise basis, using the spinal cord segmentation from the functional image. A white matter mask was derived by thresholding the PAM50 white matter probabilistic map at 0.9 in the native space, allowing separation from the gray matter.

**Fig. 3.**
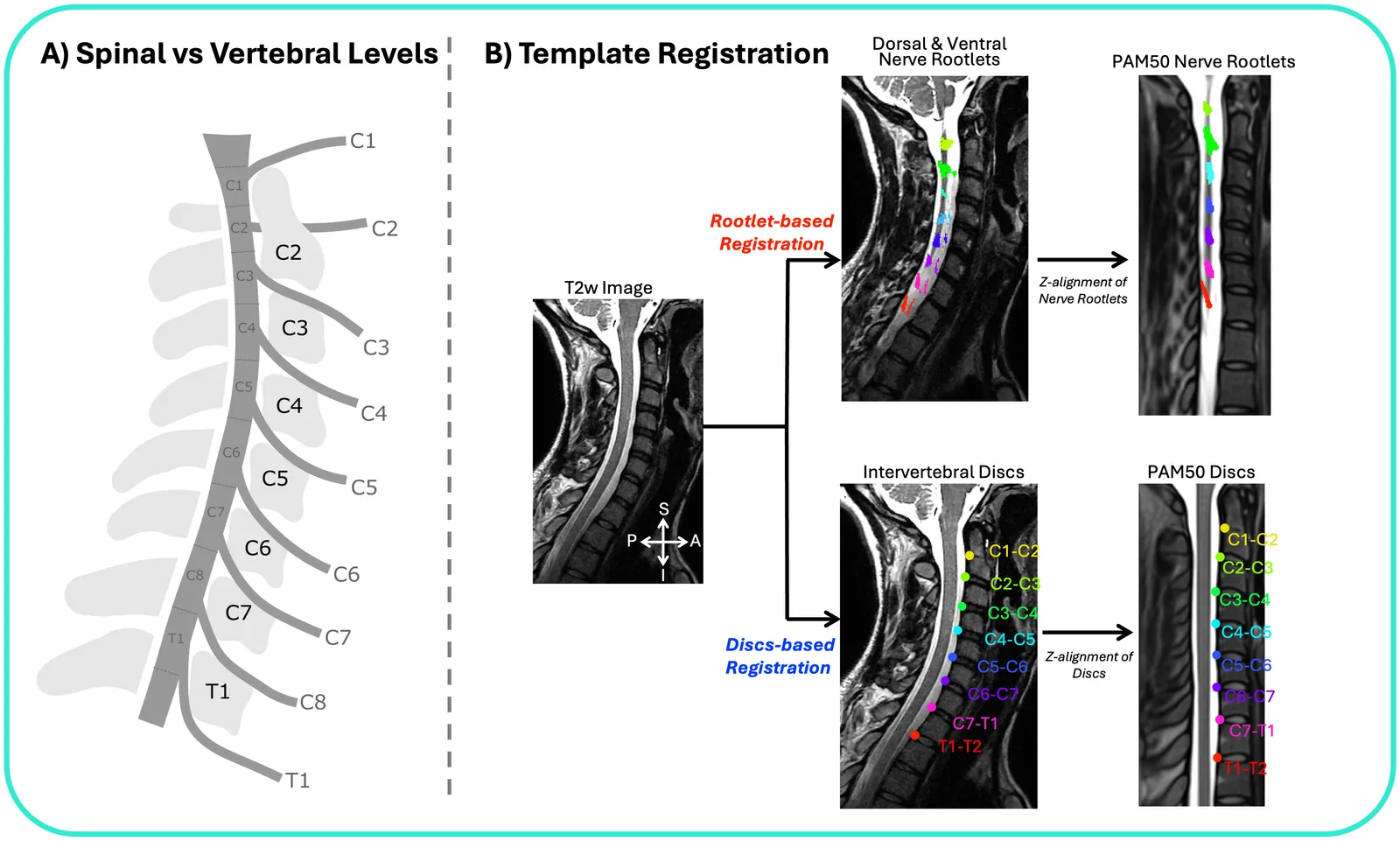
Template registration pipeline. (A) Correspondence between spinal segment levels, nerve roots, and vertebral level. (B) Registration to the PAM50 template based on nerve rootlets (red) and based on intervertebral discs (blue).

To remove physiological and motion-related noise, slicewise nuisance regressors were generated. For CSF and white matter, the first principal components of their respective signal time series were extracted using fslmeants to generate slicewise regressors. Cardiac and respiratory signals were modeled using 32 regressors derived from PNM (Brooks et al., 2008), while motion-related fluctuations were accounted for by including slicewise x-axis and y-axis translations and z-axis rotations. All nuisance regressors (n = 37) were removed using a general linear model (GLM) implemented in FSL’s FEAT. This denoising step was performed in native space because the physiological noise regressors were defined in native space, whereas subsequent spatial normalization and anisotropic smoothing were performed in template space. Slice-timing correction was applied after denoising to account for inter-slice acquisition delays.

The resulting cleaned functional time series were warped to the PAM50 template space. In this template space (straightened cord), a Gaussian smoothing kernel (FWHM: 2 mm × 2 mm × 4 mm) was applied to primarily smooth along the rostrocaudal axis of the spinal cord, an effect that would be compromised in native space due to cord curvature. Additionally, high-pass temporal filtering with a 150-second cut-off was performed. This preprocessing pipeline yields standardized, denoised functional images ready for participant-level statistical analysis.

#### 2.4.2 Statistical inference

The preprocessed fMRI time series were analyzed using a single hemodynamic response function (HRF) convolved with trialwise boxcar regressors corresponding to each stimulation intensity. A first-level activity map for each run was generated using a fixed-effects analysis with feat (FSL v6.0) and corrected for temporal autocorrelations with FILM prewhitening (Jenkinson et al., 2012). For each participant, to combine the three runs, an average activity map of each contrast of the three runs was computed using a second-level fixed-effects analysis using feat (FSL v6.0) (Jenkinson et al., 2012). Contrasts tested positive and negative signal changes, as well as linear increases and decreases across stimulation intensities. Subject-level average maps were then entered into the group-level analyses (one sample t-tests) using nonparametric permutation testing with FSL’s randomise, with a cluster-level, family-wise error (FWE) correction (Z score > 2.30, FWE p < 0.050) (Winkler et al., 2014). Age and sex were included in the group-level analyses as covariates of no interest. Analyses were restricted to the region of the spinal cord covered by each participant’s functional acquisition FOVs, which spanned approximately from C5 to T1 spinal cord levels.

Each stimulation intensity level was investigated separately, and a linear contrast across the four stimulation intensities was modeled at the group level to examine how stimulus strength modulates BOLD responses. Laterality was assessed with the left-right ratio (LR) defined by:

$$LR\;=\frac{\;L-R}{L+R}$$

With LR defined as the left-right ratio, L, the number of active voxels in the left hemicord, and R, the number of active voxels in the right hemicord.

#### 2.4.3 Region of interest analysis

Region-of-interest (ROI) analyses were performed within the spinal cord gray matter (GM) using rootlets-based registration. The GM mask was generated by binarizing the PAM50 GM atlas at a 0.5 threshold. Right and left hemicord ROIs were created by summing the corresponding PAM50 atlas regions (https://spinalcordtoolbox.com/stable/user_section/tutorials/atlas-based-analysis/adding-custom-tracts.html). ROIs were generated for the right GM, left GM, and all the spinal coverage of the group functional scans, within spinal segment levels C6, C7 and C8 from the PAM50 template (Frostell et al., 2016).

For each participant, the number of active voxels were extracted and mean Z scores were computed within each ROI for all four stimulation intensities on the subject-level average map and subject-level maps from individual runs. ROI-level statistical analyses included paired t-tests (corrected for multiple comparisons), and a linear regression (1st degree polynomial) to compare average activity within each ROI across stimulation intensities and runs. Statistical analyses were performed in Python 3.9.0 using SciPy v1.11.2 and Statsmodels v0.14.6.

#### 2.4.4 Test-retest reliability

Test-retest reliability was assessed using intra-class correlation (ICC) to quantify the stability of fMRI activity maps (Z scores) across the three runs. Voxelwise ICC was computed within the region of the spinal cord covered by the functional acquisition FOV common to all participants using a two-way mixed-effects consistency model (ICC(3,1)), to assess the consistency of fMRI responses across the three runs (Caceres et al., 2009). ICC values were interpreted according to Cicchetti’s (1994) guidelines (<0.40 poor, 0.40–0.59 fair, 0.60–0.74 good, 0.75–1.00 excellent).

To further assess replicability, the dataset was split by run, and group-level activity was analyzed for each individual run (subject-level maps from individual runs) instead of using the subject-level average map (Section 2.4.2.). This analysis was done only on the maximum stimulation amplitude (Amp4) as significant group activation was obtained only for this stimulation intensity.

#### 2.4.5 Sample size analysis

We performed a Monte Carlo-based sample size analysis to assess the robustness of group-level activity patterns. Group analyses with varying sample sizes were performed on the average of the three runs with the Amp4 stimulation intensity, using FSL *randomise* with a voxelwise threshold of Z > 2.3 and FWE cluster correction (p < 0.050). Subsamples of size 10, 15, 20, 25, 30, 35, and 40 were drawn without replacement, and each subsample analysis was repeated for 1,000 iterations.

## 3 Results

TSNR was similar across the three runs (Bonferroni-corrected paired t-test) and the average of the runs. Visual inspection suggested lower tSNR values at the most inferior spinal segment level (T1) (Fig. 4).

**Fig. 4.**
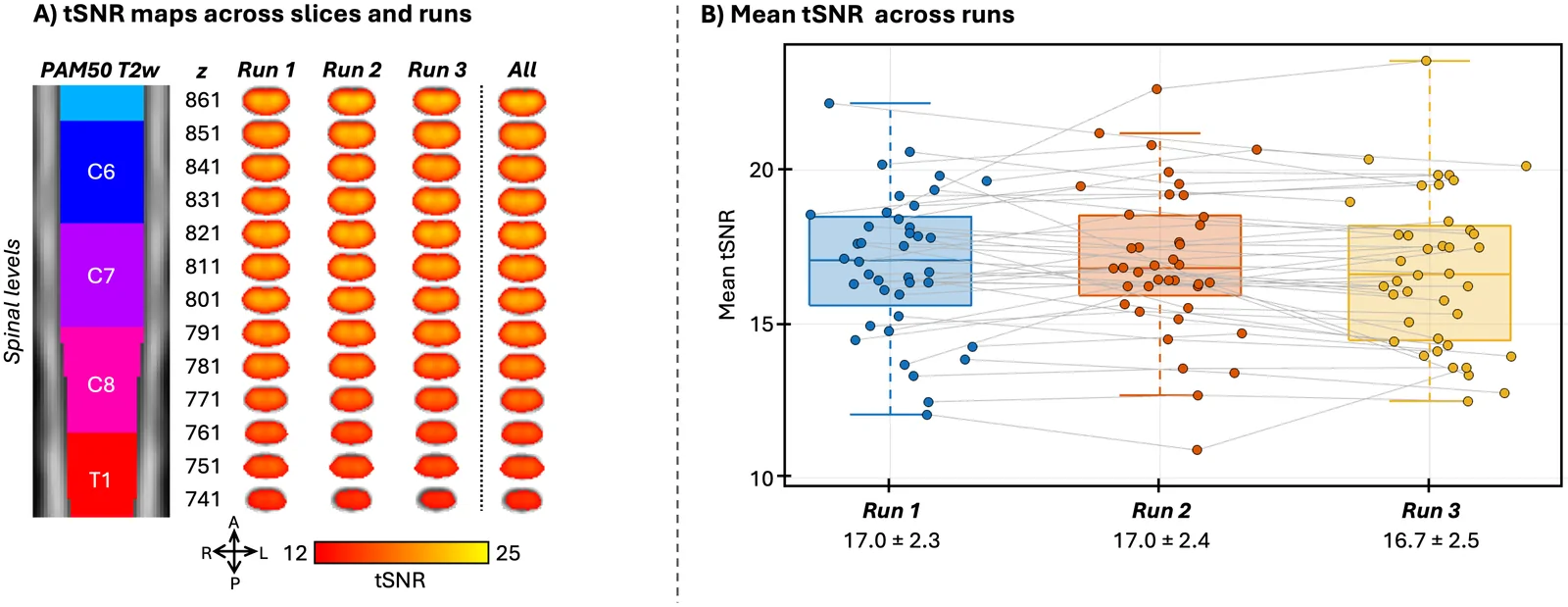
Temporal signal-to-noise ratio (tSNR) after motion correction. (A) tSNR map in the PAM50 template space for each run and across all runs after motion correction. The mean column represents the average tSNR within the spinal cord mask, across all runs. Rootlets-based spatial normalization was used to generate the group level tSNR map. tSNR was computed in the native space, after motion correction (linear interpolation) for each run, and warped to the template with linear interpolation. Group tSNR is similar across the 3 runs, while it decreases at the inferior field-of-view. (B) Mean tSNR across runs after motion correction in the native space. Each dot represents one participant. Mean tSNR and standard deviation are reported under each run. No significant differences were found across runs (Bonferroni-corrected paired t-test). Boxplots show median, 25th and 75th percentile, brackets show 1.5 times interquartile range.

### 3.1 Impact of anatomical landmark for registration

We investigated the impact of anatomical landmarks on functional group activity maps using either spinal nerve rootlets or intervertebral discs labels during template registration (see Fig. 3). A mean T2w image (N = 40) registered to the template shows sharper delineation of the rootlets when using rootlets-based compared to disc-based registration (see Supplementary Fig. S1). For the functional results, group-level maps for Amp4 comparing both rootlet and discs-based registration are presented in Figure 5. Rootlet-based registration yielded more spatially localized activation clusters at C7 compared to disc-based registration. The average Z score within the GM was significantly higher for rootlet-based registration (2.78 ± 0.39) than for disc-based registration (2.53 ± 0.26; paired Wilcoxon signed-rank test, p < 0.001). In contrast, the number of active voxels within the GM did not differ significantly between methods (rootlets: 399 vs discs: 374; proportion test, p > 0.05). The same improvement was found for the linear contrast (see Supplementary Fig. S2).

**Fig. 5.**
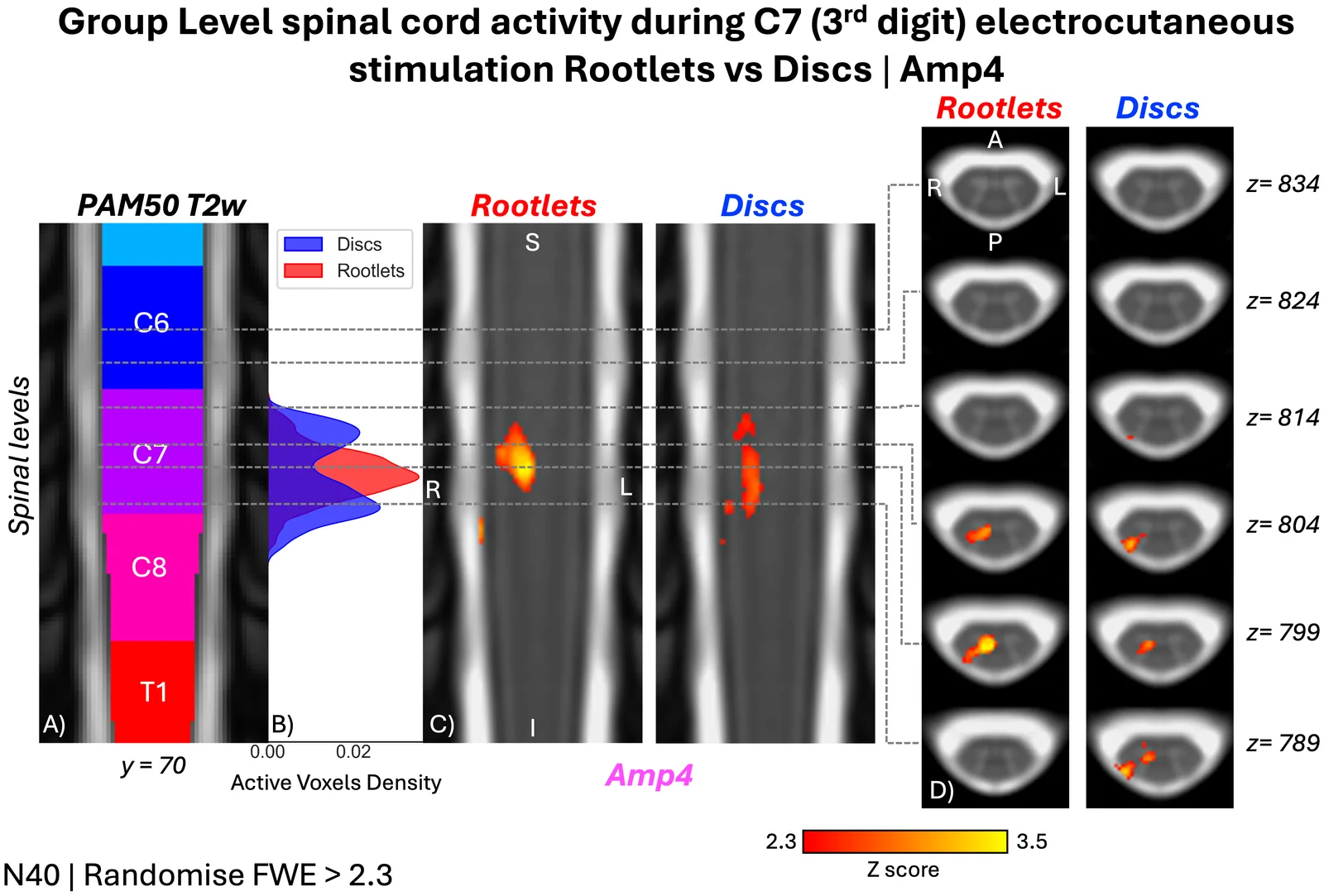
Group-level activity maps during electrocutaneous stimulation of the 3rd digit of the right hand (N = 40, Amp4) comparing rootlets- (red) vs. discs-based (blue) registration. (A) Coronal view (y = 70, PAM50) with spinal segment levels. (B) Active voxel density plot. (C) Coronal slices with activity overlay. (D) Axial views of representative clusters. Maps: Randomise, Z score > 2.3, and cluster corrected for family-wise error (FWE) at p < 0.050. In the coronal view, clusters appear narrower with a higher Z score, whereas the disc is more spread out.

### 3.2 Stimulation intensity

For the lowest stimulation intensity (Amp1), with an average amplitude of 2.4 ± 0.9 mA, the reported pain intensity was 0.2 ± 0.5. For the maximum stimulation amplitude of 6.5 ± 3.0 mA (Amp4), the mean reported pain intensity was 4.4 ± 2.8. High-intensity pain ratings were significantly higher than low-intensity ratings (paired Wilcoxon signed-rank test, p < 0.001).

#### 3.2.1 Intensity encoding at the participant level

At the participant level, the BOLD signal increased significantly with stimulation intensity in the right dorsal horn, particularly at C6–C7 spinal segment levels, as reflected by greater number of active voxels and higher average Z scores across intensities, and significant linear fit (Fig. 6). No significant increase was found in the number of active voxels or in the average Z score across intensities in the left GM.

**Fig. 6.**
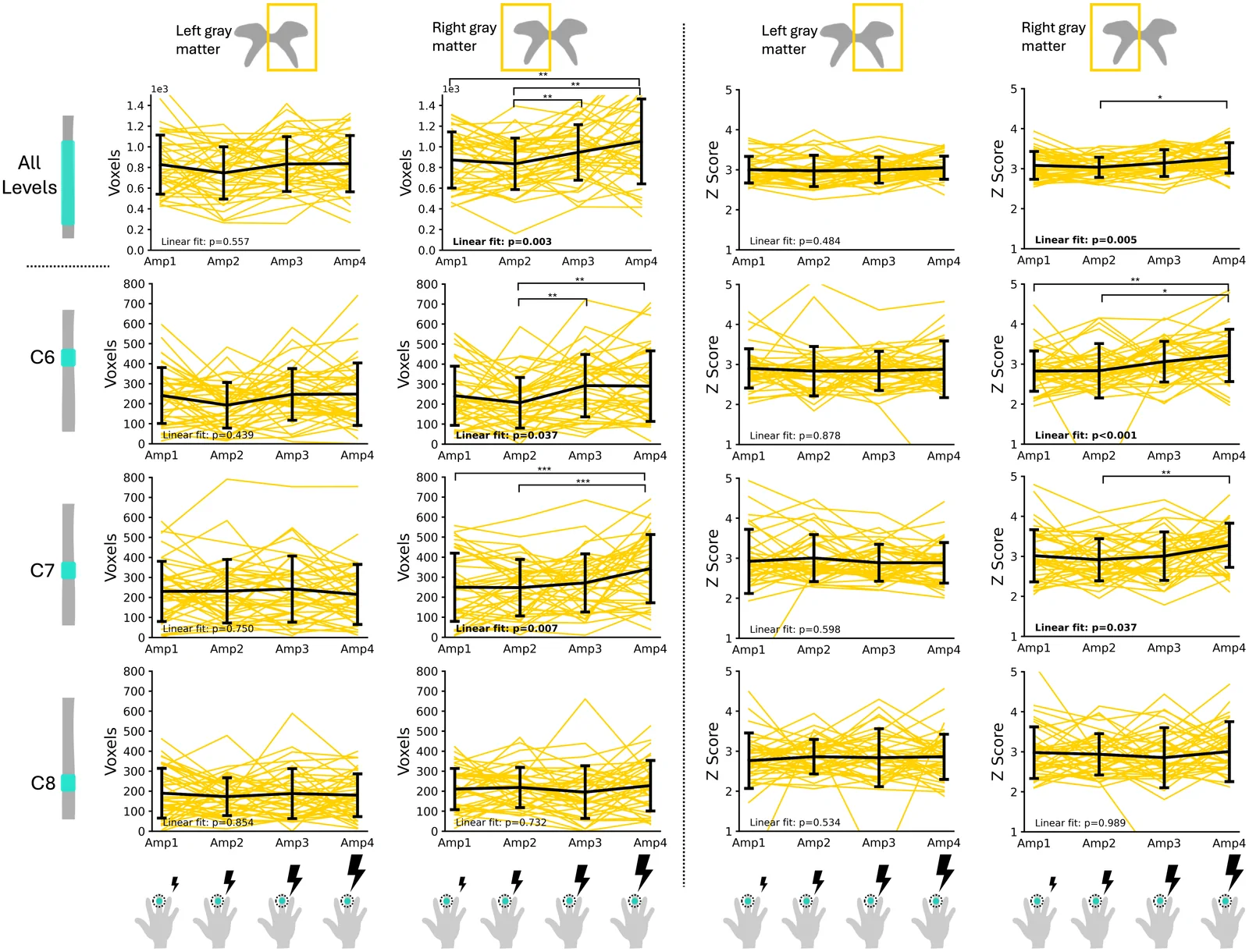
Participant-level active voxels and average Z score across stimulation intensities. Number of active voxels and average Z score within left and right spinal cord gray matter across stimulation intensities for all spinal segment levels, and specifically C6, C7, and C8 (p < 0.050). Yellow lines represent one individual participant; the black line shows the group average, with brackets indicating standard deviations. Significance: *p < 0.050, **p < 0.010, ***p < 0.001 (Bonferroni-corrected paired t-test). A significant linear increase of active voxels and average Z score is observed in the right gray matter across all spinal segment levels, and specifically at C6 and C7. Black lines show the mean; brackets standard deviation.

#### 3.2.2 Intensity encoding at the group level

All group level results are shown using rootlets-based registration methods, or mentioned explicitly if otherwise.

At the group level, the maximum intensity (Amp4) showed significant clusters of activity localized to the right dorsal horn at C7 spinal segment level (Z score > 2.3; FWE p < 0.050; Fig. 7). Intensities Amp1, Amp2, and Amp3 did not yield any significant clusters that passed FWE correction. No significant negative BOLD responses or linear decreases in signal were observed across stimulation intensities. Uncorrected maps for each stimulation intensity are provided in the Supplementary Figure S3. In addition, the BOLD signal increased linearly with stimulation intensity, localized to the caudal end of C6 and C7 spinal segment levels and the right dorsal horn (i.e., negative LR ratio) (Fig. 7). No relationship between sex, age, and spinal activity was found. We also provide a group-level map using threshold-free cluster enhancement (TFCE; Supplementary Fig. S5).

**Fig. 7.**
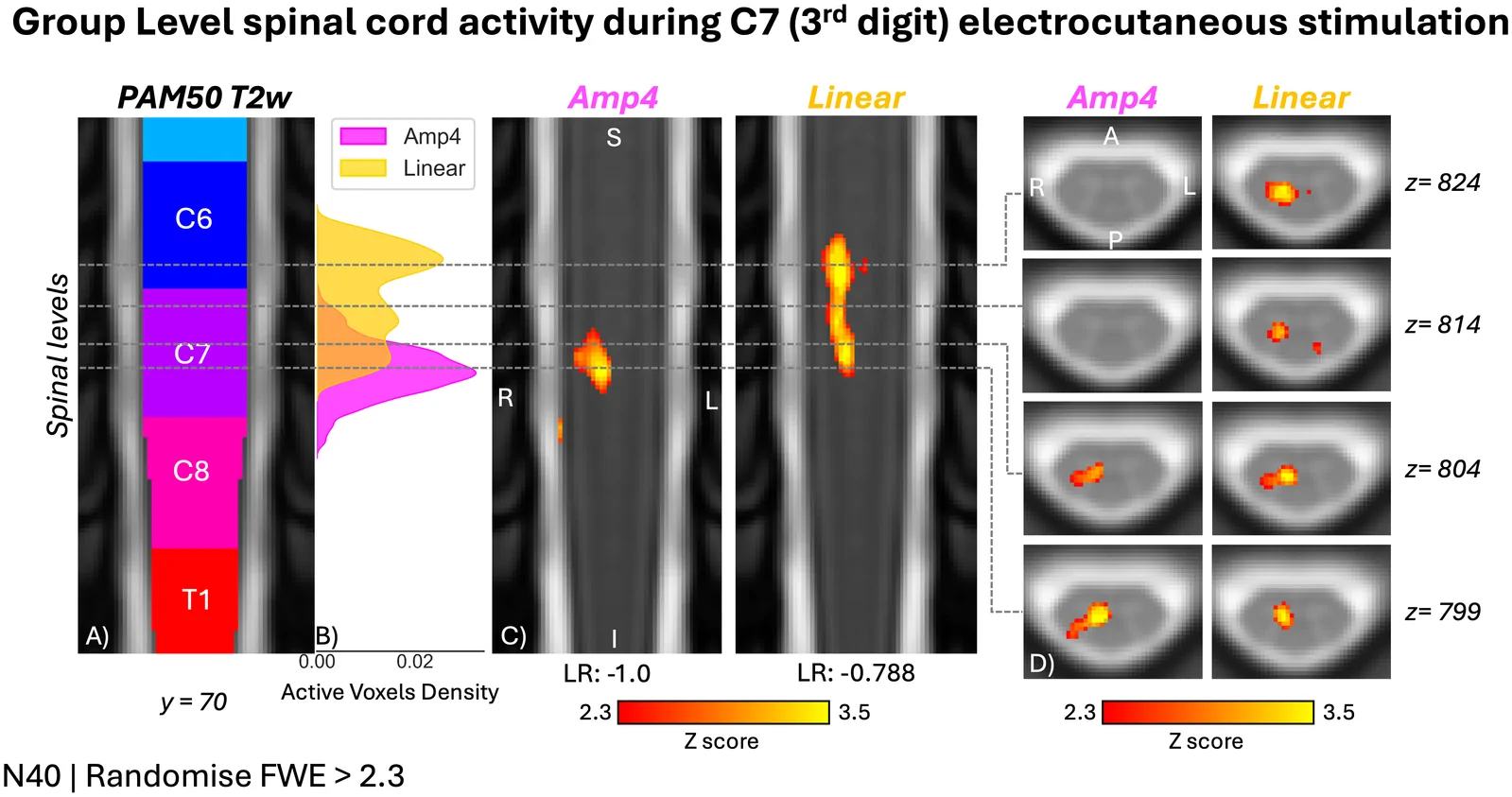
Group-level activity maps during electrocutaneous stimulation of the 3rd digit of the right hand (N = 40) with various stimulation intensities. (A) Coronal view (y = 70 on the PAM50 referential system) with an overlay of the spinal segment levels, matching the coverage of the functional EPI scans. (B) Active voxel density for stimulation intensity Amp4 and linear fit between stimulation intensities. (C) Coronal slices with an overlay of the activity maps. The location of the activity was assessed using the left-right (LR) ratio. (D) Axial views of representative activity clusters. The activity maps were generated using a voxelwise thresholded at Z score > 2.3, and cluster corrected for family-wise error (FWE) at p < 0.050. Simulation amplitudes Amp1, Amp2, and Amp3 did not yield any significant clusters that passed FWE correction, and are not shown here; uncorrected maps for each stimulation intensity are provided in the Supplementary Figure S3. Rootlets-based spatial normalization was used to generate the group level maps.

### 3.3 Test-retest reliability

As stimulation intensities Amp1, Amp2, and Amp3 did not yield any significant clusters at the group level, we mainly focus on stimulation intensity Amp4 for the subsequent analysis to investigate the test-retest reliability across runs. All group-level results are shown using rootlets-based registration methods, or mentioned explicitly if otherwise.

#### 3.3.1 Intra-class-correlation across runs

We first report voxelwise ICC across the three runs and participants for stimulation intensity Amp4 (Fig. 8). ICC was generally low, but showed a higher ICC localized at C7 spinal segment level, in the right dorsal horn around 0.5, which corresponds to fair reliability (Cicchetti, 1994).

**Fig. 8.**
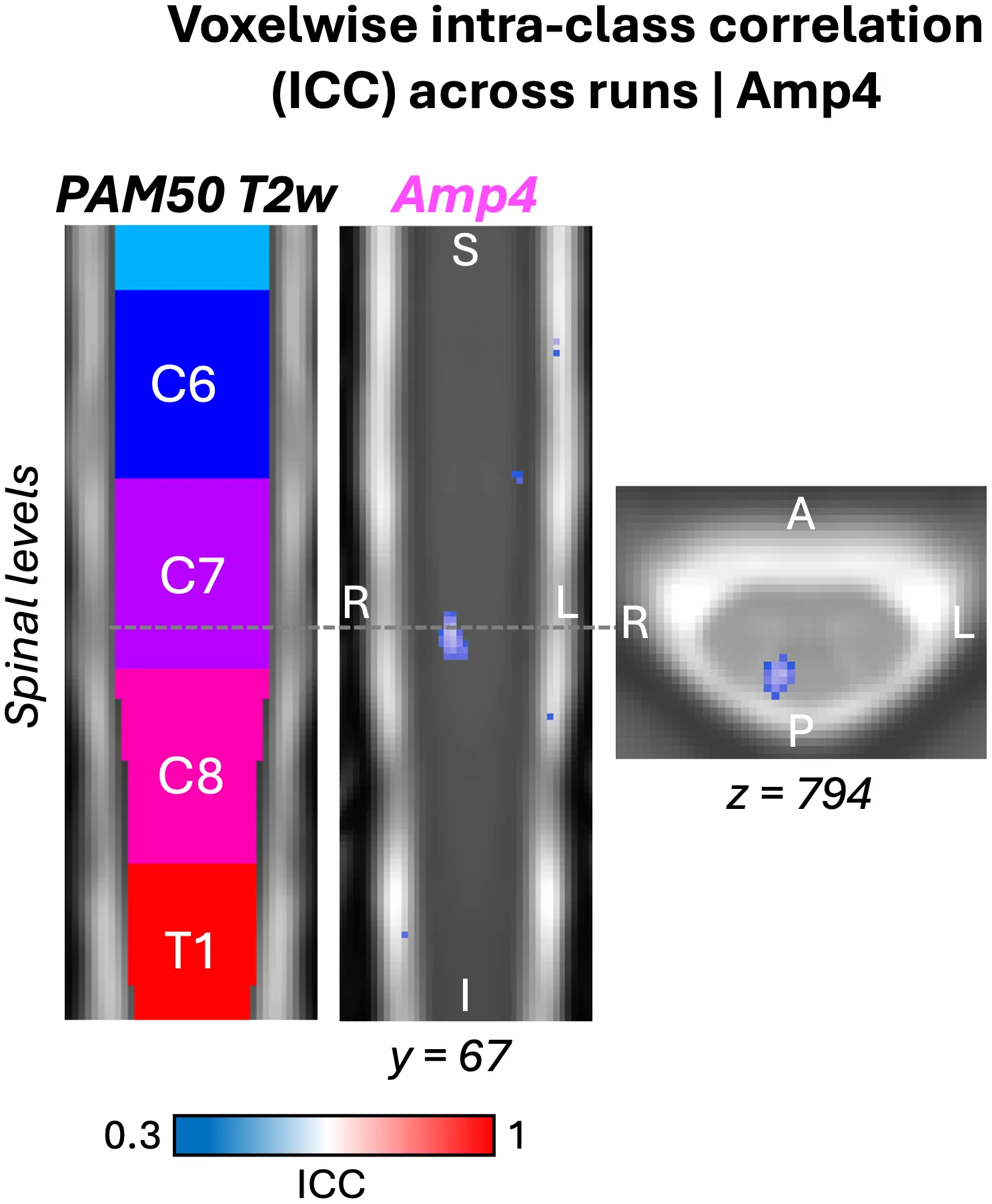
Voxelwise intra-class correlation (ICC) across the three runs and participants for stimulation intensity Amp4. The PAM50 template with overlaid spinal segment levels is shown on the left. A coronal slice (y = 67) with the corresponding ICC map is displayed in the center, and an axial slice (z = 794) with ICC overlay is shown on the right. Higher ICC values indicate greater consistency of activity across runs. Group-level ICC maps were generated using rootlet-based spatial normalization on the activity maps (Z score). ICC(3,1) was computed quantifying the consistency of activity across the three runs and participants.

#### 3.3.2 Participant-level analysis across runs

To investigate the low reliability across runs, we examined Amp4 responses at the participant level by quantifying the number of active voxel and average Z score within the GM across spinal segment levels for each of the three runs. As shown in Figure 9, the number of active voxels decreased linearly across runs at all segment levels, whereas average Z scores did not change significantly. To determine whether this decrease of active voxels emerged within a single run, we also assessed active voxel counts and average Z scores across trials in run-1 (see Supplementary Fig. S6) and found no significant within-run trend. Potential confounds were evaluated and did not support alternative explanations: motion outliers, did not increase across runs (Supplementary Fig. S7), and maximum reported pain intensity was stable across runs (Supplementary Fig. S8).

**Fig. 9.**
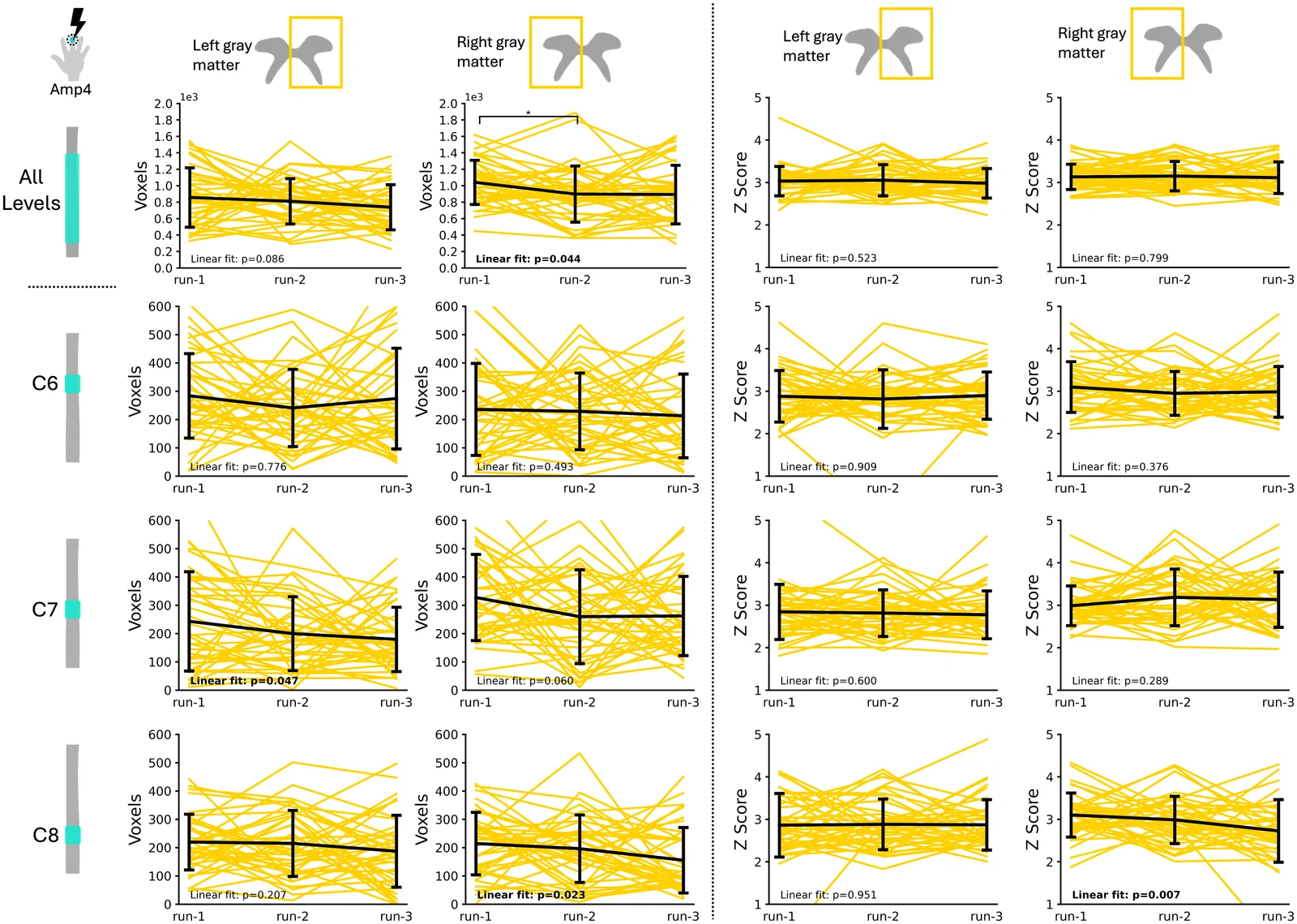
Average number of active voxels and average Z score within left and right spinal cord gray matter across runs for Amp4 and all spinal segment levels, C6, C7 and C8. Each yellow line represents one participant as the black line represents the average across all participants with the brackets showing the standard deviation. Bonferonni-corrected paired t-test. *: p < 0.050; **: p < 0.010; ***: p < 0.001. The number of active voxels decreases between runs across all levels and at C7 spinal segment level. The average Z score did not change significantly across runs. Black lines show the mean; brackets standard deviation.

#### 3.3.3 Group-level analysis across runs

We next examined group-level activity separately for each run. Run-1 showed significant clusters for Amp3, Amp4, and linear contrast (Supplementary Fig. S9; randomise, Z score > 2.3, FWE-corrected p < 0.050), matching the pattern obtained when averaging across all three runs (see Supplementary Fig. S10). In contrast, runs 2 and 3 did not produce any FWE-corrected significant clusters for any intensity. Uncorrected maps (Fig. 10), nevertheless, suggest a reduction in the Amp4 response after run-1, with fewer active voxels and lower Z score in runs 2 and 3, consistent with the density distributions of active-voxel. We also observe this same reduction across runs when using disc-based registration (Supplementary Fig. S4); while run 1 produced significant clusters, runs 2 and 3 did not yield any FWE-corrected significant clusters for any stimulation intensity.

**Fig. 10.**
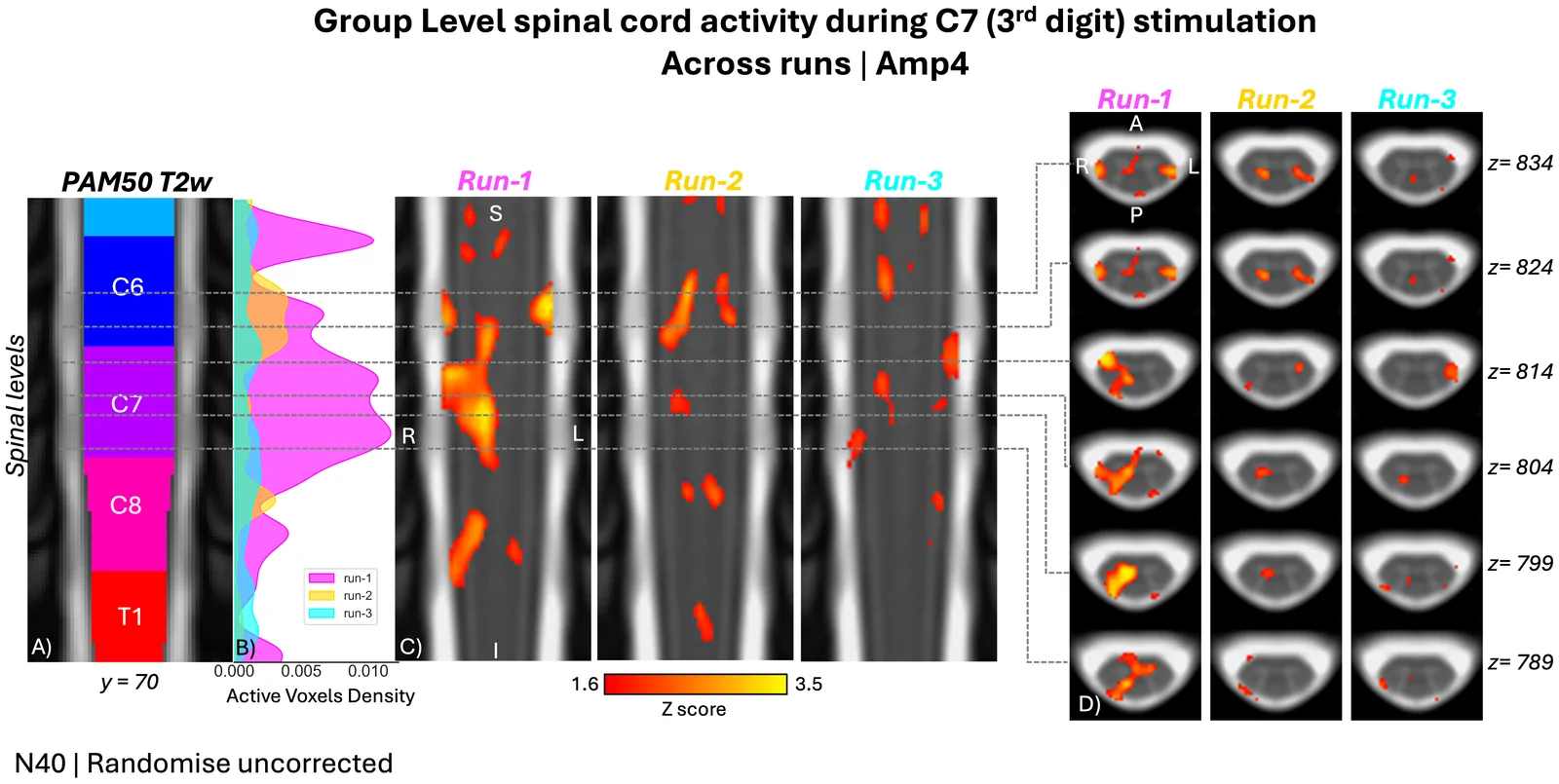
Group-level activity maps during electrocutaneous stimulation of the 3rd digit of the right hand (N = 40) with Amp4 across individual runs. (A) Coronal view (y = 70 on the PAM50 referential system) with an overlay of the spinal segment levels, matching the coverage of the functional EPI scans. (B) Density plot of active voxels for stimulation amplitude Amp4 across three runs. (C) Coronal slices with an overlay of the activity maps. (D) Selected axial views centered on representative activity blobs. The activity maps were generated using a Randomise model, voxelwise thresholded at Z score > 1.6, and uncorrected for family-wise error (FWE). Rootlets-based spatial normalization was used to generate the group-level maps. A lower density of active voxels is observed between run-1 vs. run-2 and run-3.

### 3.4 Sample size

The Monte Carlo analysis provided an empirical estimate of the sample size required to reliably detect segment-specific activity (Amp4, across all runs). We observed that the activity cluster at C7 spinal segment level was detected above 50% of the time with a sample size of N = 28 (Supplementary Fig. S11). By repeatedly resampling the dataset across increasing group sizes, we observed a clear improvement in detection frequency with larger cohorts. It is important to note that, since there were 1,000 iterations, there were some repetitions of sample for higher sample size.

## 4 Discussion

Our findings identify practical conditions under which segment-level spinal sensory responses can be detected with spinal cord fMRI. Across participants, electrocutaneous stimulation of the third digit of the right hand was most consistently localized near the C7 spinal cord segment. Detectability increased with stimulation intensity. Critically, group inference was improved by anatomical normalization that incorporated rootlet landmarks, supporting the view that functionally relevant anchors can reduce artifactual spread introduced by disc-based alignment. At the same time, responses attenuated across repeated runs, indicating that repetition can materially alter measured effects and should be explicitly modeled or mitigated in the study design. Together, these results help reconcile prior reports of both focal and distributed activation and provide a framework for more reliable spinal fMRI studies of human sensory processing.

### 4.1 Impact of anatomical landmark: Disc vs. nerve rootlet reference

An important step in mapping dermatomes with fMRI is the choice of anatomical landmarks used during spatial normalization (Kinany et al., 2023). Aligning participants by vertebral or disc levels can decrease the reliability of group-level maps because levels based on spine anatomy are an indirect proxy for the anatomy of interest, the spinal segment levels. In contrast, normalizing data using spinal nerve rootlets better aligns corresponding spinal segments across participants and therefore improves functional correspondence, as reflected by increased average Z score and more localized clusters of activity in the group-level maps (Fig. 5).

In previous work, we reported that rootlet-based registration improved alignment compared with disc-based registration in a task-based motor fMRI study (Bédard, Valošek, et al., 2025). However, motor tasks typically recruit multiple myotomes and span several spinal segments, producing spatially broad activation. As a result, it was difficult to determine whether the improved registration truly enhanced segment-level localization, since the “ground truth” activation was not expected to be focal. Here, using a localized sensory stimulus that engages a more restricted segmental representation, we can demonstrate a clearer improvement in group-level localization.

### 4.2 Mapping of dermatomes with electrocutaneous stimulation

#### 4.2.1 Spatial localization

At both the participant and group levels, we observed a linear increase of BOLD responses with higher stimulation intensities, primarily localized to the right GM at the C7 spinal segment level and extending partially to the caudal end of C6 (Figs. 6 and 7). This localization is consistent with the expected dermatome (3rd digit stimulation, C7 dermatome). The activity extending to C6 spinal segment level could be associated with the fact that afferents entering via the C7 nerve root do not necessarily synapse at the C7 spinal cord segment; fibers can ascend or descend several levels within Lissauer’s tract before terminating in the dorsal horn (Coggeshall et al., 1981; Light & Perl, 1979; Sugiura et al., 1986). We did not observe a comparable intensity-dependent response at the C8 spinal segment level, which could be due to the lower tSNR measured at C8 (Fig. 4), where proximity to the lungs increases field inhomogeneity, susceptibility artifacts, and signal loss (Finsterbusch, 2014).

In the present study, the use of rootlet-based spatial normalization likely improved segment-level correspondence across participants, which may have contributed to the more localized group-level activity observed here around C7 compared to previous studies. For example, Stracke et al. (2005) used electrical stimulation targeting the C6, C7, and C8 dermatomes at 1.5T and observed activity not only at corresponding levels but also at C3 and C4, which was attributed to the potential involvement of propriospinal interneurons. More generally, Landelle et al. (2021) noted in a review that spinal fMRI activity often peaks at the expected dermatome level but typically spans multiple segments. Consistent with this, Weber et al. (2020) applied tactile stimulation to the lateral shoulder (C5) and the dorsal surface of the third digit (C7), and observed activity extending across multiple segments in both conditions. Similarly, Lawrence et al. (2008) used vibration across several dermatomes (C5, C7, C8, T1, L4, S1) and found peak activity at expected levels alongside spread to adjacent segments and bilateral dorsal and ventral activity. Differences across studies may also reflect the stimulation site and intensity; however, because modalities vary (electrocutaneous, tactile, vibration), stimulus intensity is difficult to compare directly. Some of the apparent rostrocaudal spread reported in prior work may also reflect methodological factors, particularly the use of disc-based alignment as a proxy for spinal segments. Because vertebral levels do not map consistently onto spinal cord segments across individuals, imperfect segmental alignment at the group level can blur responses and produce artificially widespread activity.

Axially, both the maximum stimulation intensity (Amp4) and linear-contrast clusters were localized to the anterior portion of the dorsal horn, consistent with expected sensory pathways. Aβ afferents terminate primarily within the intermediate dorsal horn laminae (i.e., laminae IV), which aligns with the observed activity pattern (Fig. 7). However, the limited axial resolution and the use of in-plane spatial smoothing likely restricted our ability to resolve layer-specific effects. Recent 7T work by Horn et al. (2025) demonstrated layer-specific activity to noxious heat, highlighting the potential to distinguish fiber-specific projections within the GM with higher-resolution ultra-high field spinal imaging.

High lateralization to the right hemicord was found for Amp4 and for the linear contrast (Fig. 7), consistent with stimulation of the right hand and with previous studies (Weber et al., 2016a, 2016b). We did not observe increasingly bilateral responses with higher intensity. This is in line with Brooks et al. (2012) who reported ipsilateral responses for both thermal innocuous and noxious stimulation. However, this contrasts with studies reporting more bilateral spinal cord activity for noxious stimulation, such as Summers et al. (2010) using noxious laser stimuli, and Weber et al. (2016a) who found bilateral responses (along with ventral horn involvement and rostrocaudal spread from C4–C8) during both innocuous and painful heat stimulation of the C6 dermatome.

#### 4.2.2 Intensity encoding

When examining stimulation intensities separately, group-level analyses (randomise, Z > 2.3, FWE p < 0.050) revealed no significant activity for Amp1, Amp2, or Amp3, although Amp3 yielded significant clusters within run-1. In contrast, Amp4 produced significant activity localized to the right GM at the C7 spinal segment level.

Because the experiment included four stimulation intensities, the number of stimulation blocks contributing to each individual intensity was reduced. As a result, statistical power was lower for each intensity level, particularly for Amp1 and Amp2. Therefore, the absence of significant activation at these lower intensities should not necessarily be interpreted as an absence of underlying neural responses. In addition, stimulation intensities were individualized using a thresholding procedure designed to define an individualized stimulation range rather than precisely estimate perceived sensory or pain intensity. Consequently, variability in the perceived intensity associated with a given stimulation level (e.g., Amp1–Amp3) may have further reduced sensitivity to detect consistent group-level effects. Because pain ratings were not collected separately for each stimulation intensity during fMRI acquisition, we were unable to directly assess the relationship between perceived intensity and BOLD responses for each stimulation level. Future studies may benefit from increasing the number of repetitions per intensity, obtaining intensity-specific perceptual ratings, or focusing on fewer stimulation conditions.

The detection of significant activity only at Amp4 is, nevertheless, consistent with the expectation that increasing stimulation amplitude increases effective afferent drive, thereby producing stronger BOLD responses. In our study, Amp4 was also frequently perceived as painful during thresholding, suggesting that the Amp4 effect may reflect, at least in part, the transition from innocuous to noxious stimulation: 6 out of 40 participants reported Amp1 as painful compared to 35 out of 40 for Amp4. Consistent with this interpretation, Summers et al. (2010) reported stronger spinal BOLD responses and more bilateral activity for noxious thermal stimulation compared with innocuous brushing.

Although participants tolerated the stimulation during fMRI acquisition, the use of different stimulation durations during thresholding and imaging may have contributed to variability in subjective perception of the stimulation.

### 4.3 Reliability across runs

We explored the reliability of the BOLD activity across runs and found that for Amp4, reliability at the C7 segment was moderate (ICC ≈ 0.5; Fig. 8), noting that estimating ICC across three runs can yield lower values than pairwise comparisons.

The reliability observed in the present study is broadly consistent with previous spinal fMRI literature. Resting-state studies have demonstrated reproducible dorsal–dorsal and ventral–ventral connectivity patterns, with reliability generally ranging from fair to good, although reliability decreases substantially at the voxel and single-segment levels (Barry et al., 2016; Kaptan et al., 2023; O. S. Kowalczyk et al., 2024). Similarly, task-based spinal fMRI studies have reported anatomically plausible and reproducible activation patterns but often only poor-to-fair reliability of activation magnitude, particularly when assessed voxelwise or across sessions (Dabbagh et al., 2024; O. Kowalczyk et al., 2025). Across both resting-state and task-based paradigms, reliability tends to improve when signals are averaged within anatomically defined regions and when optimized denoising strategies are employed (Hu et al., 2018; O. S. Kowalczyk et al., 2024). Taken together, these findings suggest that spinal fMRI can reliably identify the spatial organization of neural activity, whereas the reliability of activation magnitude remains more modest and sensitive to methodological factors. Our finding of moderate reliability across runs is, therefore, consistent with the current state of the spinal fMRI literature.

To understand this limited repeatability, we examined each run separately. At the participant level, the number of active voxels decreased significantly from run-1 to runs 2 and 3 across all segment levels within the right GM (Fig. 9), whereas mean Z scores did not change. This suggests that the reduction across runs was primarily driven by a decrease in the spatial extent of activation rather than a decrease in activation magnitude within the remaining active voxels. At the group level, run-1 produced significant clusters (Amp3, Amp4, and the linear contrast; Supplementary Fig. S9), but no FWE-corrected clusters were detected in runs 2 or 3 for any intensity; the same trend was observed when using disc-based registration Supplementary Figure S4. Importantly, we did not observe a comparable decline within run-1 across trials (Supplementary Fig. S6), suggesting that the effect emerged primarily between runs rather than within a run.

This decrease in signal across runs could reflect habituation or adaptation of the participant to the electrical stimulation. Although maximum pain ratings did not change significantly across runs, they trended downward (Supplementary Fig. S8). However, we did not systematically investigate participants’ perception of the stimulation, and the maximum tolerable intensity was not necessarily perceived as painful by all participants. Furthermore, pain ratings were only collected at the end of each fMRI run and were not recorded separately for each individual stimulation intensity. As such, the ratings reflected the combined experience of all stimulation intensities and showed substantial inter-subject variability (Supplementary Fig. S8). Consequently, the relationship between habituation, subjective perception, and the observed decrease in signal across runs cannot be fully assessed. Increased head/neck motion is an alternative explanation for reduced reliability across runs. However, the number of motion outliers, defined using DVARS, did not increase across runs (Supplementary Fig. S7). While DVARS reflects signal intensity changes and may capture sources of noise beyond motion, and may be less sensitive to gradual movements, the absence of an increase in outliers suggests that abrupt motion-related artifacts were unlikely to differ across runs.

Evidence for habituation/adaptation to electrical stimulation in spinal cord fMRI is limited. Kündig et al. (2025) reported substantially reduced detected activity across runs and during a second session of lumbosacral spinal cord fMRI using non-noxious tibial nerve stimulation. Habituation to repeated electrical stimuli is also well documented in brain fMRI, particularly for noxious stimulation, where activity in pain-related regions decreases over repeated noxious electric stimuli (Nickel et al., 2014). Paul et al. (2021) showed that habituation patterns differed for innocuous versus noxious stimulation of the hand, with more generalized somatosensory habituation compared to pain-specific regions.

Habituation/adaptation likely contributed to the reduced run-to-run repeatability observed here, but its physiological origin remains uncertain. As electrocutaneous stimulation bypasses mechanoreceptors, peripheral receptor adaptation is unlikely (Graczyk et al., 2018; Johnson, 2007). Adaptation of the peripheral nerve level is also improbable under the parameters used, as peripheral nerve fatigue typically requires higher-frequency stimulation (200 Hz) than was applied here (Graczyk et al., 2018). More plausible explanations include reduced excitability at the first synapse in the dorsal horn (e.g., presynaptic inhibition or other short-term plasticity mechanisms) and/or increased descending modulation from supraspinal regions, both of which could contribute to the observed reduction in activity across runs. Prior work also suggests that habituation can build over time, leading to progressively faster signal attenuation in subsequent runs, which is consistent with our observations (Higashiyama & Tashiro, 1989).

Several methodological factors may have accelerated habituation/adaptation in this experiment, including testing four stimulation amplitudes within the same session and using a fixed inter-run rest interval. In addition, the thresholding procedure was performed during the same session as the fMRI experiment, which could also have contributed to habituation/adaptation. Higashiyama and Tashiro (1989) investigated adaptation to electrocutaneous stimulation across levels spanning sensation to pain thresholds and reported greater adaptation at lower pulse amplitudes, with slower adaptation at higher amplitudes. In our study, because four amplitude levels were presented in randomized order, the lower amplitudes may have contributed to faster overall adaptation or habituation. Similar results were reported by Buma et al. (2007) although adaptation there was quantified perceptually rather than using fMRI (Buma et al., 2007; Higashiyama & Tashiro, 1989).

Future studies may incorporate longer and/or randomized rest periods, consider limiting the number of amplitude conditions per session, and re-evaluate stimulation parameters such as pulse width, frequency, and waveform (monophasic versus biphasic; anodal versus cathodal) to reduce the effects of habituation (Herrero et al., 2000; A. Y. J. Szeto & Mao, 1982). For example, A. Y. Szeto and Saunders (1982) reported greater adaptation at higher stimulation frequencies, and Kaczmarek (2000) and A. Y. J. Szeto and Mao (1982) found that 15–30 minutes rest periods allowed recovery from adaptation. In addition, while many neurophysiological protocols set intensity relative to the detection threshold, some paradigms increase intensity across repeated runs to counteract habituation, which may be worth testing in future spinal fMRI work (Nierula et al., 2024).

In contrast to the decrease in activity across runs observed here, Weber et al. (2016a) reported evidence of sensitization in the spinal cord during painful thermal stimuli related by an increased spatial extent of the activity across runs, which was not the case with the non-painful heat stimulus.

### 4.4 Sample size

The Monte Carlo analysis provides practical guidance for future sensory spinal cord fMRI studies. We estimated that a sample size of approximately 28 participants was required to detect C7 activity in more than 50% of resampling iterations for the strongest stimulation condition (Amp4). Detection frequency increased progressively with larger sample sizes, highlighting the importance of adequate statistical power for identifying segment-specific activity. These findings are consistent with previous spinal cord fMRI studies, which have shown that increasing both sample size and the length of the time series data improves activation detectability and spatial localization (Hemmerling et al., 2023; Weber et al., 2016b). Given that spinal cord fMRI studies have historically used relatively modest sample sizes due to the technical challenges (Kinany et al., 2023), these results provide useful guidance for the design of future sensory spinal cord fMRI experiments.

### 4.5 Limitations and future work

This study has several limitations. Although the cohort was balanced for sex (20 females, 20 males), the study was not designed or powered to investigate biological effects such as sex or age. Our sample-size analysis indicated that n = 28 participants were required to reliably detect C7 activity (Supplementary Fig. S11), leaving limited power for subgroup analyses. Future studies with larger cohorts may be better positioned to evaluate potential demographic influences on spinal cord responses to sensory stimulation.

Several methodological aspects of spinal cord fMRI remain under active investigation. There is no standardized spinal fMRI preprocessing pipeline, and choices related to spatial smoothing, nuisance regression, physiological noise correction, and statistical inference may influence activity maps and complicate comparisons across studies (Grajauskas et al., 2019; Hemmerling et al., 2023). For example, the use of white matter nuisance regression remains debated, as partial-volume effects may remove variance associated with nearby GM activity and potentially affect sensitivity to neural signals (Grajauskas et al., 2019). Similarly, the optimal approach to statistical inference remains uncertain. In addition, using a two-step GLM may not be fully equivalent to simultaneous modeling and may influence the variance structure, particularly if the task and nuisance regressors share variance. Although cluster-level inference is widely used in fMRI, the assumptions underlying spatial clustering may be challenged in spinal cord fMRI due to the small size and geometry of the cord, sharp tissue boundaries, and residual physiological noise. To assess the robustness of our findings, we repeated the analyses using TFCE (Smith & Nichols, 2009), which yielded similar activation patterns (Supplementary Fig. S5). Nevertheless, future studies may benefit from further evaluation of statistical inference methods specifically optimized for spinal cord fMRI data.

Another methodological consideration is the use of a single canonical HRF. The optimal spinal cord HRF remains uncertain and may vary across participants, spinal segments, and tissue types, as it has been demonstrated in the brain (Giulietti et al., 2008; Handwerker et al., 2004; Li et al., 2019). Future work should investigate whether more flexible or data-driven HRF models improve sensitivity and spatial specificity in spinal cord fMRI analyses.

The present study targeted a single dermatome. Mapping multiple dermatomes will be necessary to determine whether stimulation of adjacent sites produces cross-dermatomal effects (e.g., habituation) and to test the generalizability of our findings. In addition, robust segment-level localization was most evident at higher stimulation intensities and in the initial run, with clear attenuation across repeated runs. This run-related reduction in detectability, consistent with habituation/adaptation and/or other session-dependent factors, could bias multi-run paradigms if not explicitly modeled or mitigated.

Stimulation intensities were individualized to account for inter-subject differences in sensory sensitivity. While this approach improved tolerability and enabled stimulation over a comparable perceptual range across participants, it limited our ability to directly relate activation magnitude to absolute stimulus intensity or to establish quantitative dose–response relationships. Several limitations related to the thresholding procedure should be acknowledged. Stimulation intensities were determined using a single ascending series designed to identify an individualized range extending from sensory threshold to the maximum tolerable electrical stimulation intensity, rather than to precisely estimate sensory or pain thresholds. Thresholding was performed using 2 seconds stimulations, whereas 5 seconds stimulations were delivered during fMRI acquisition, which may have influenced subjective perception of some stimulation levels. Furthermore, pain ratings were collected only at the end of each fMRI run and, therefore, did not permit assessment of individual stimulation intensities or their relationship to the observed fMRI responses.

Finally, the present findings are specific to electrocutaneous stimulation and may not generalize to other forms of somatosensory input. Different stimulation modalities recruit peripheral afferents through distinct mechanisms and have been shown to produce different patterns of neural activation in the brain (Alouit et al., 2025).

In our next study, we will address these questions by examining sex effects, multiple dermatome sites, neural injury, and preprocessing steps in a larger cohort of healthy participants and in patients with cervical radiculopathy.

## 5 Conclusion

We characterized spinal cord sensory processing within the spinal cord with electrocutaneous stimulation during fMRI. Spinal activity increased with stimulation intensity and was primarily localized within the right dorsal horn at the expected C7 spinal segment level. Importantly, rootlet-based spatial normalization improved segmental alignment across participants, yielding greater spatial specificity and sensitivity than conventional disc-based normalization. Responses attenuated across repeated runs, consistent with habituation/adaptation that can reduce detectability in multi-run paradigms if not modeled. Together, these findings support the use of spinal fMRI to directly examine how sensory signals are organized and distributed within the human spinal cord, and provide a methodological foundation for more accurate localization of cervical sensory processing in basic research and translational research.

## Supplementary Material

Supplementary Material

## Data Availability

The code for preprocessing and data analysis is available on GitHub: https://github.com/kennethaweberii/Dermatomal_Mapping_R01/releases/tag/r20260213. The data is accessible at https://doi.org/10.18112/openneuro.ds008784.v1.0.0.

## Footnotes

1
https://ksfoundationepic.org/Documentation/html/index.html
