# Supplementary material for: Spatial distribution of spinal cord fMRI activity with electrocutaneous stimulation
Source: Imaging Neurosci (Camb). 2026 Oct 1;4:IMAG.a.1383. doi: 10.1162/IMAG.a.1383 (PMC13636297; doi:10.1162/IMAG.a.1383)
Supplement: Supplementary file 1 [file IMAG.a.1383_supp.pdf]

12. Supplementary material

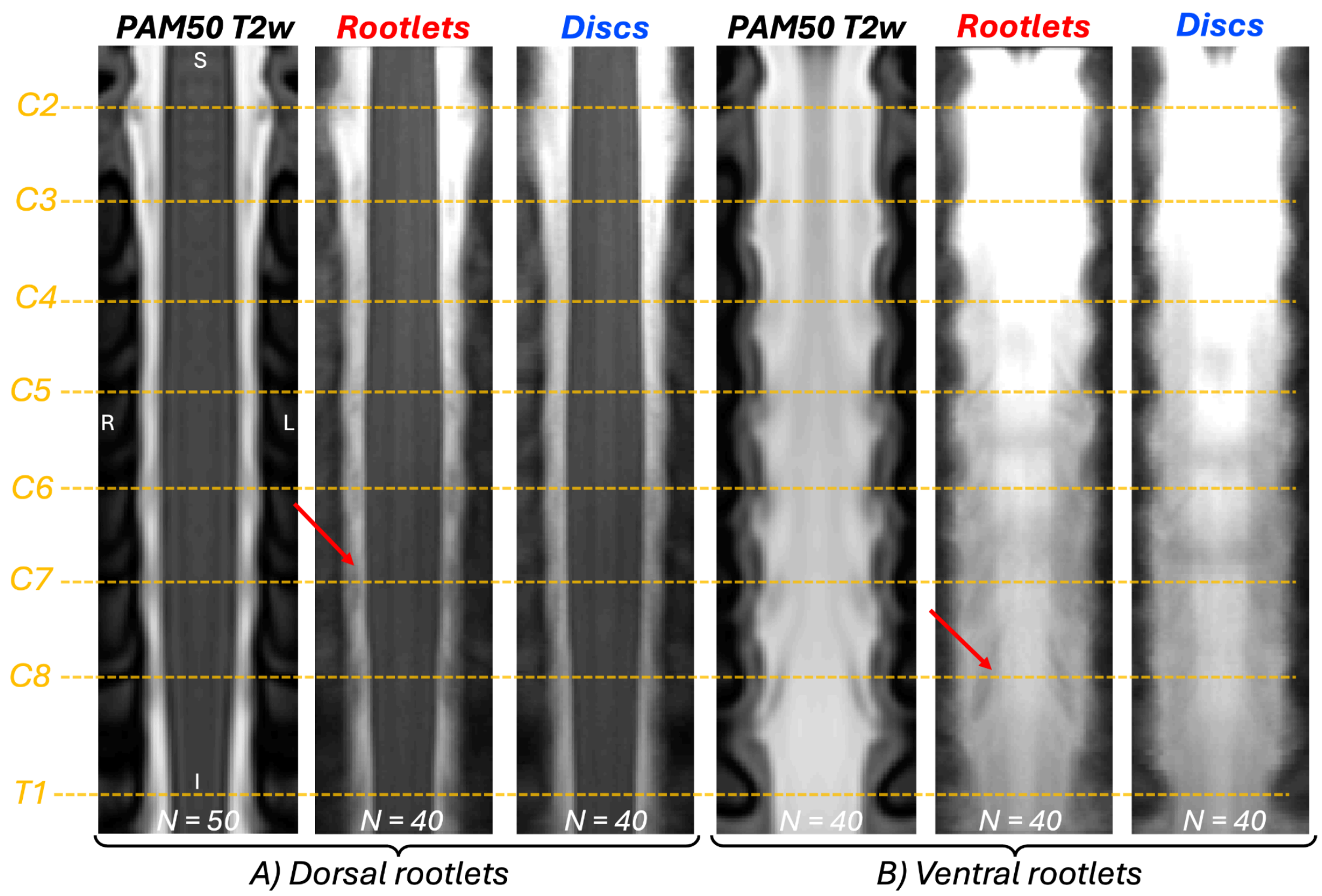

Figure S1. *Group* ***Mean T2w image (N = 40) registered to the PAM50 template using either rootlet-based (red) or disc-based (blue) spatial normalization.*** *The PAM50 T2-weighted template image is shown for reference (black). Orange dashed lines mark the centers of spinal cord segments C2–T1 as defined in PAM50.* ***A)*** *Representative coronal slice (y = 70) illustrating dorsal rootlets.* ***B)*** *Representative coronal slice (y = 79) illustrating ventral rootlets. Red arrows indicate examples of well-delineated rootlets. Overall, rootlets are sharper and show better correspondence across participants with rootlet-based normalization than with disc-based normalization.*

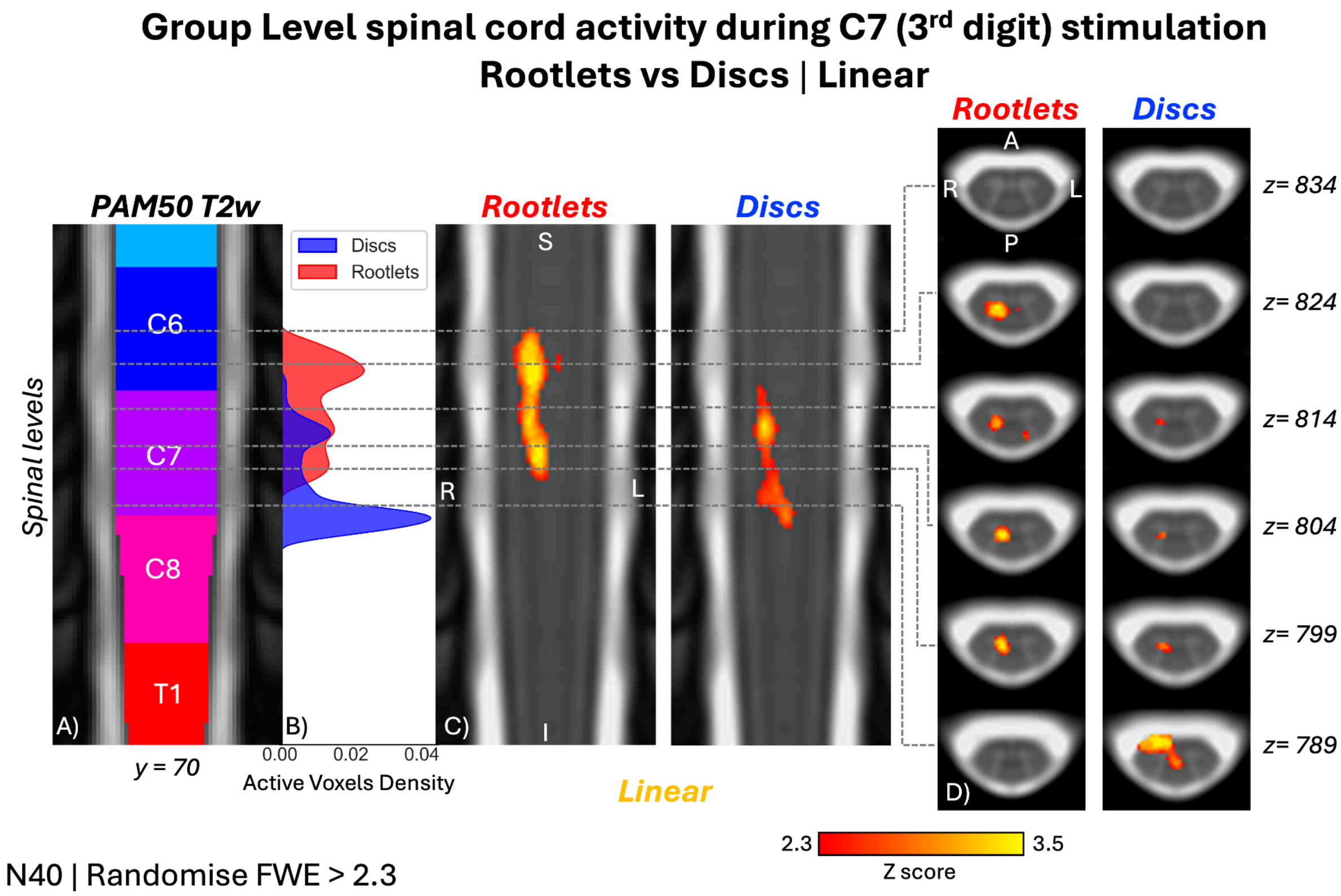

Figure S2. ***Group-level activity maps during electrocutaneous stimulation of the 3rd digit of the right hand (N = 40, Linear) comparing rootlets- (red) vs. discs-based (blue) normalization.*** ***A)*** *Coronal view (y = 70, PAM50) with spinal segment levels.* ***B)*** *Active voxel density.* ***C)*** *Coronal slices with activity overlay.* ***D)*** *Axial views of representative clusters. Maps: Randomise, Z score > 2.3, and cluster corrected for family-wise error (FWE) at p < 0.050. In the coronal view, the cluster appears narrower and shows a higher Z score, whereas the disc shows less active voxels and lower Z score.*

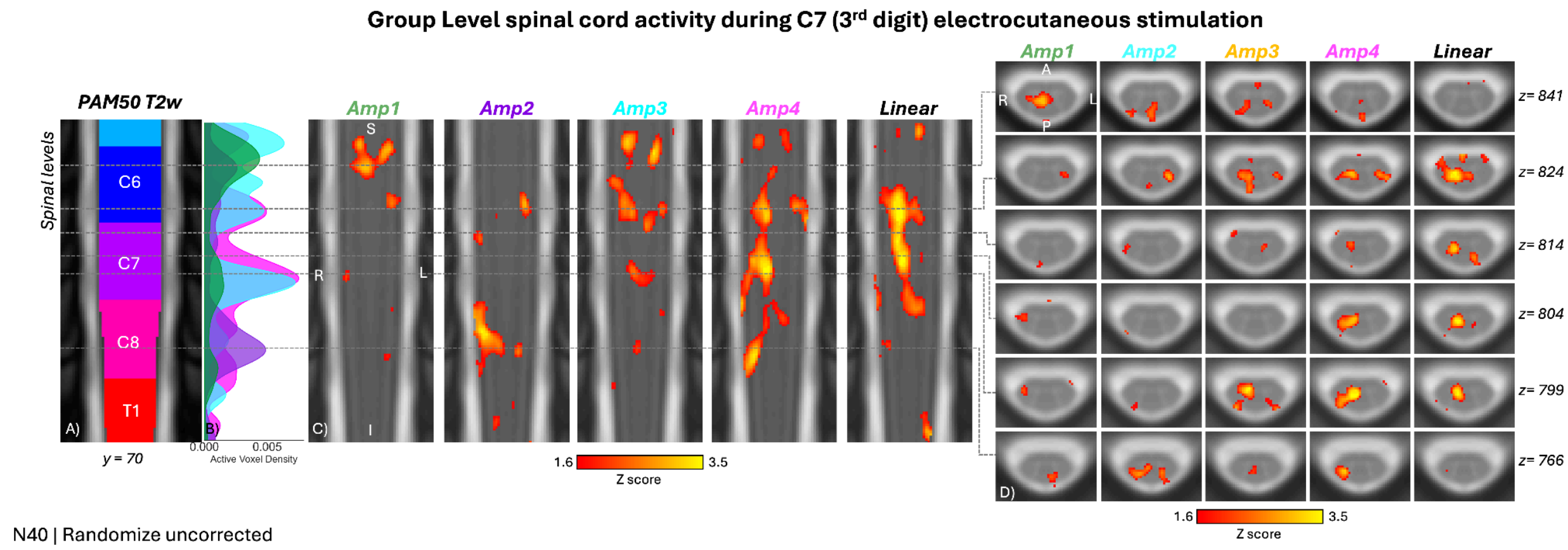

Figure S3. *Group-level activity maps during electrocutaneous stimulation of the 3rd digit of the right hand (N = 40) with various stimulation amplitudes.* ***A)*** *Coronal view (y=70 on the PAM50 referential system) with an overlay of the spinal segment levels, matching the coverage of the functional EPI scans.* ***B)*** *Density plot of active voxels for stimulation amplitude Amp4 and linear fit between stimulation intensities.* ***C)*** *Coronal slices with an overlay of the activity maps. The location of the activity was assessed using the left-right (LR) ratio.* ***D)*** *Selected axial views centered on representative activity blobs. The activity maps were generated using a Randomise model, voxelwise thresholded at Z score > 1.6, and uncorrected for family-wise-error.*

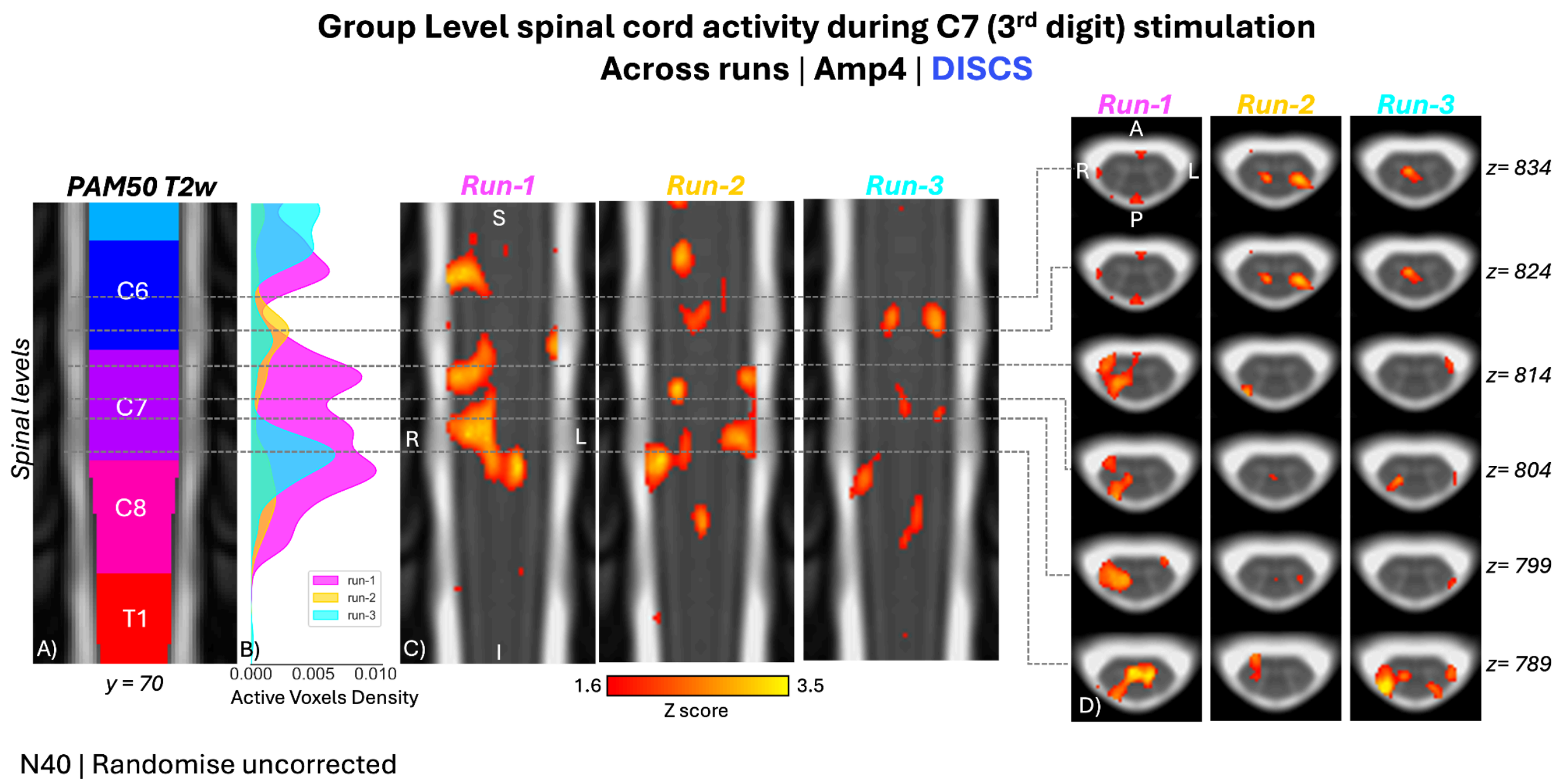

Figure S4. ***Group-level activity maps during electrocutaneous stimulation of the 3rd digit of the right hand (N = 40) with Amp4 across individual runs using disc-based registration.*** ***A)*** *Coronal view (y=70 on the PAM50 referential system) with an overlay of the spinal segment levels, matching the coverage of the functional EPI scans.* ***B)*** *Density plot of active voxels for stimulation amplitude Amp4 across three runs.* ***C)*** *Coronal slices with an overlay of the activity maps.* ***D)*** *Selected axial views centered on representative activity blobs. The activity maps were generated using a Randomise model, voxelwise thresholded at Z score > 1.6, and uncorrected for family-wise error (FWE). A lower density of active voxels is observed between run-1 vs run-2 and run-3. FWE-maps did not yield any significant clusters for runs 2 and 3.*

Group Level spinal cord activity during C7 (3rd digit) electrocutaneous stimulation

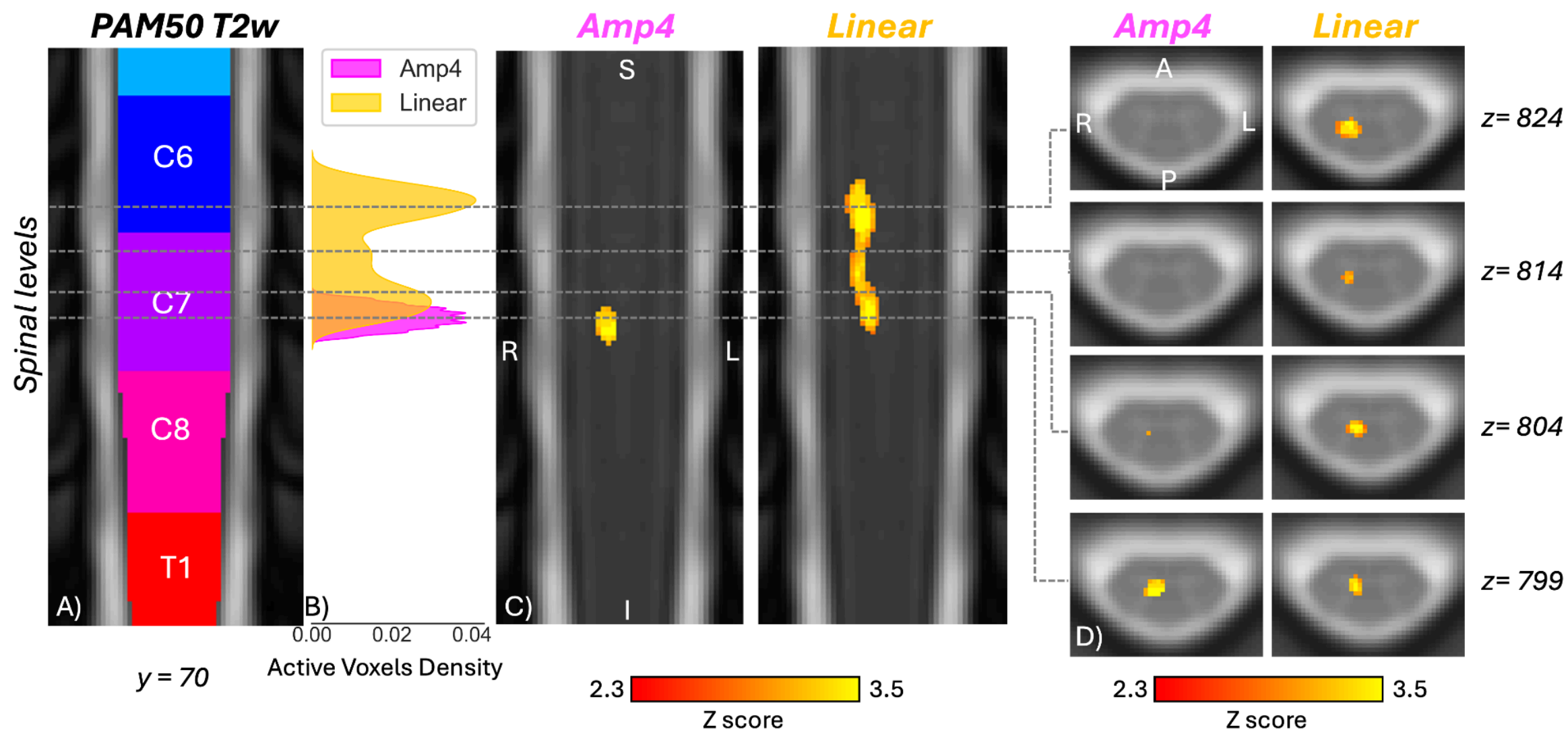

N40 | Randomise TFCE

Figure S5. ***Group-level activity maps during electrocutaneous stimulation of the 3rd digit of the right hand (N = 40) with various stimulation intensities.*** ***A)*** *Coronal view (y = 70 on the PAM50 referential system) with an overlay of the spinal segment levels, matching the coverage of the functional EPI scans.* ***B)*** *Active voxel density for stimulation intensity Amp4 and linear fit between stimulation intensities.* ***C)*** *Coronal slices with an overlay of the activity maps.* ***D)*** *Axial views of representative activity clusters. The activity maps are reported with threshold-free cluster enhancement (TFCE) correction and $p < 0.05$, and were generated with rootlets-based registration. Simulation amplitudes Amp1, Amp2 and Amp3 did not yield any significant clusters.*

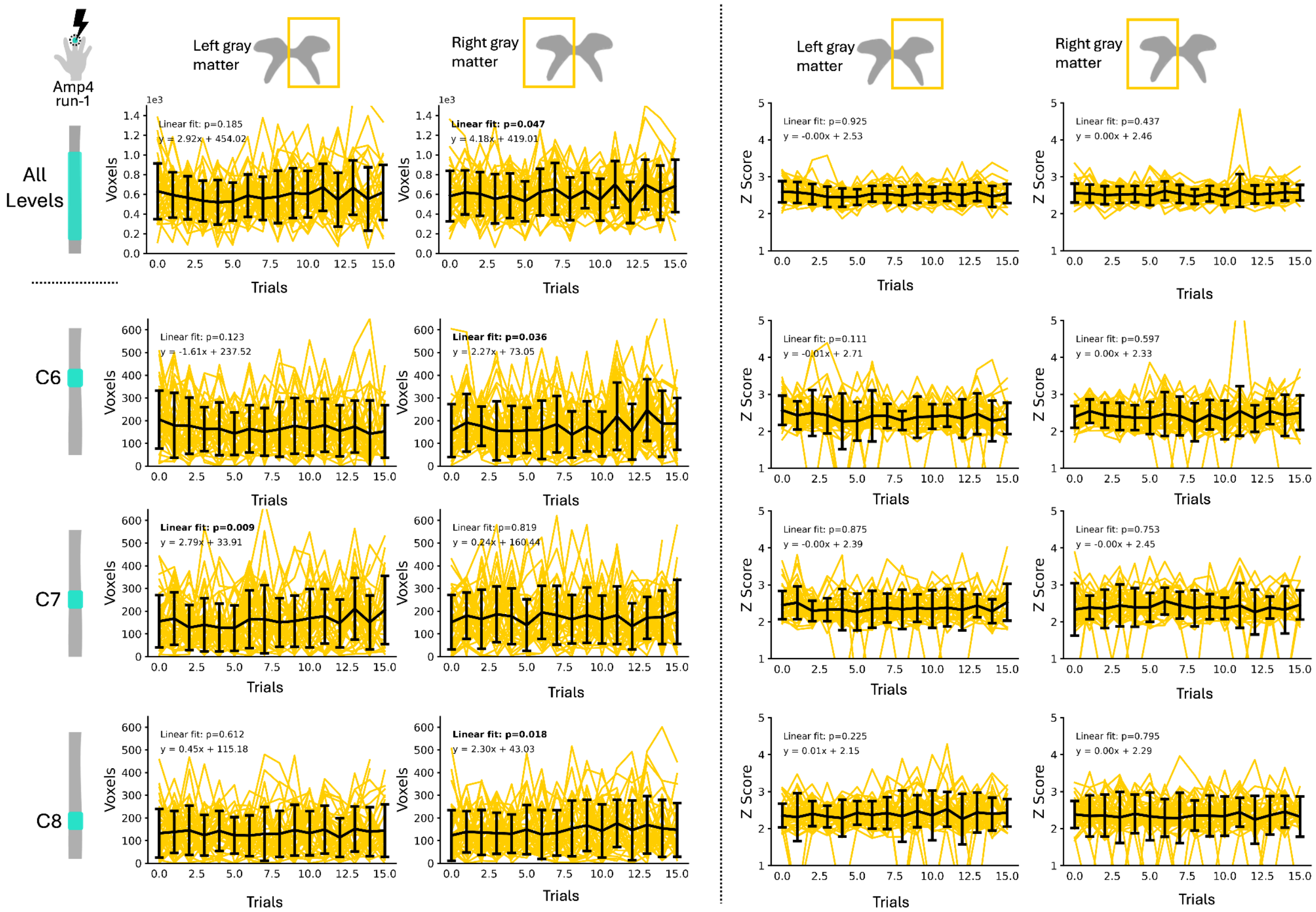

Figure S6. Average number of active voxels and Z score within left and right spinal cord gray matter within run 1 for intensity Amp4 for each stimulation trial ($p < 0.050$) for all spinal segment levels, C6, C7 and C8. *Each yellow line represents one participant as the black line represents the average across all participants with the brackets showing the standard deviation. The number of active and average Z scores stay constant across trials. Linear fits are also shown. Black lines show the mean; brackets standard deviation*

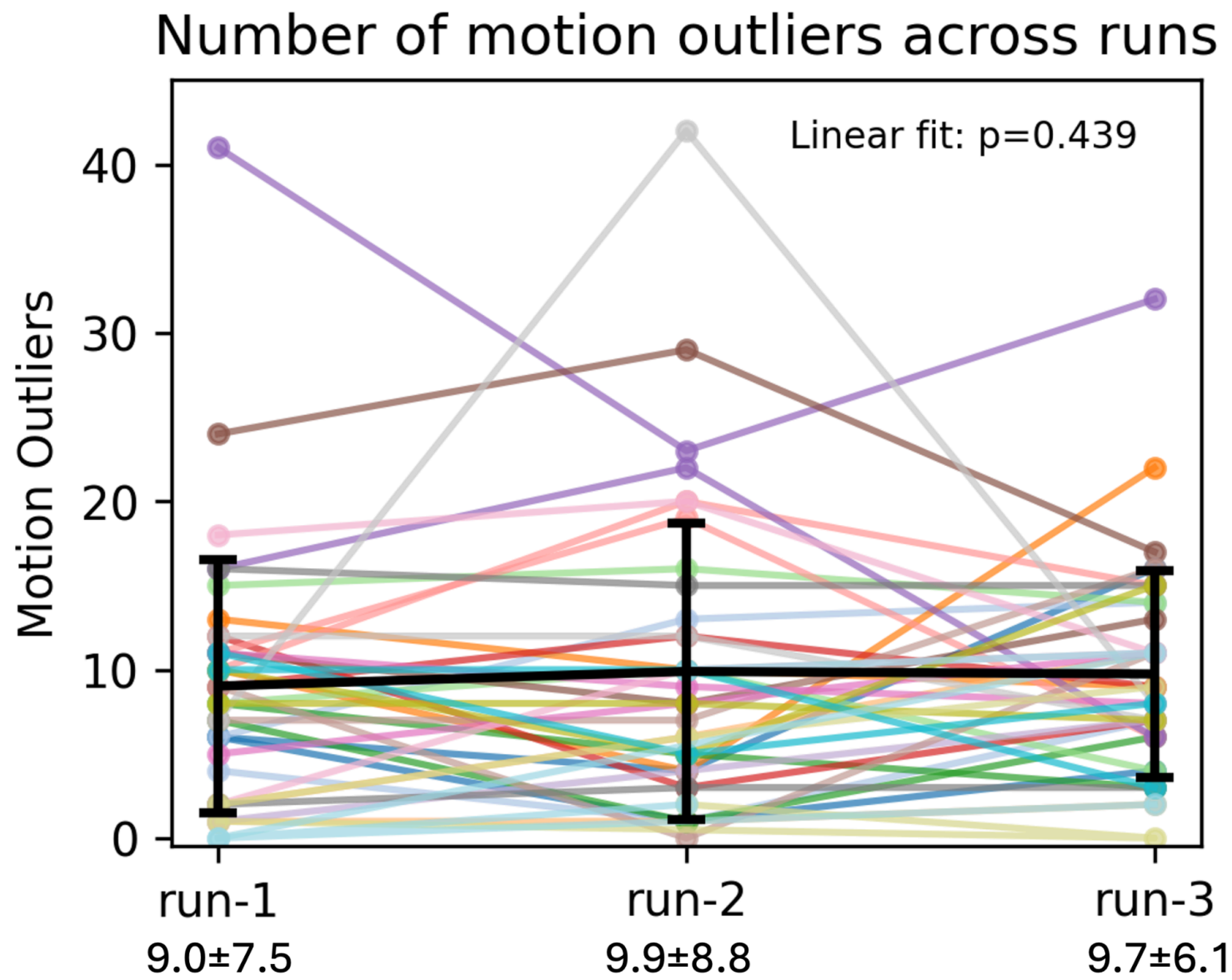

Figure S7. **Number of motion outliers across participants and runs.** *Each line represents one participant as the black line represents the average across all participants with the brackets showing the standard deviation with values under each run (376 volumes per run). Motion outliers were measured based on DVARS which corresponds to the root mean square of the temporal change of the fMRI voxelwise signal at each time point. The threshold is defined with the 75th percentile + 1.5 times the interquartile range. Black lines show the mean; brackets standard deviation*

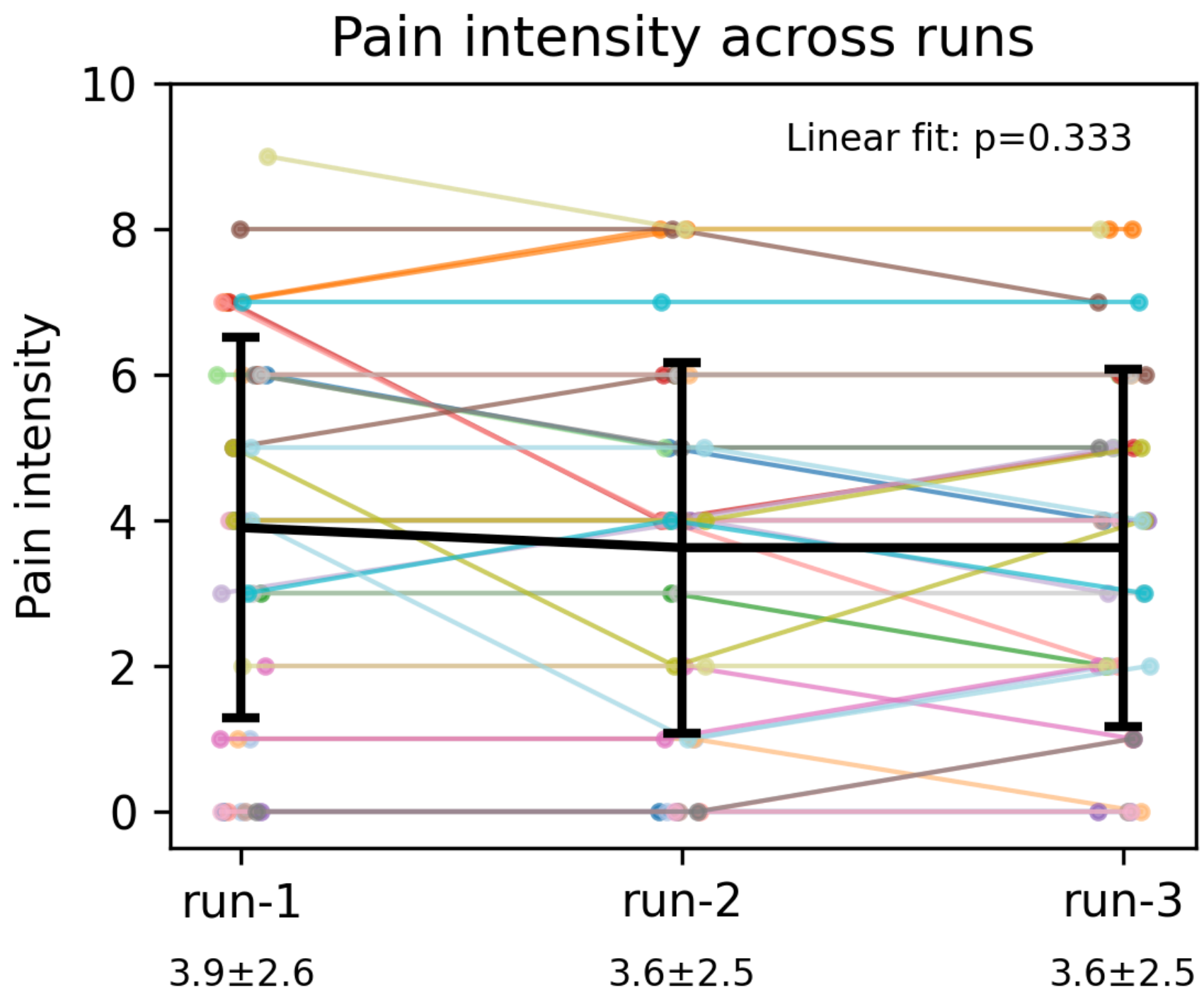

Figure S8. Maximum pain intensity across participants and runs. *Each line represents one participant as the black line represents the average across all participants with the brackets showing the standard deviation with values under each run.*

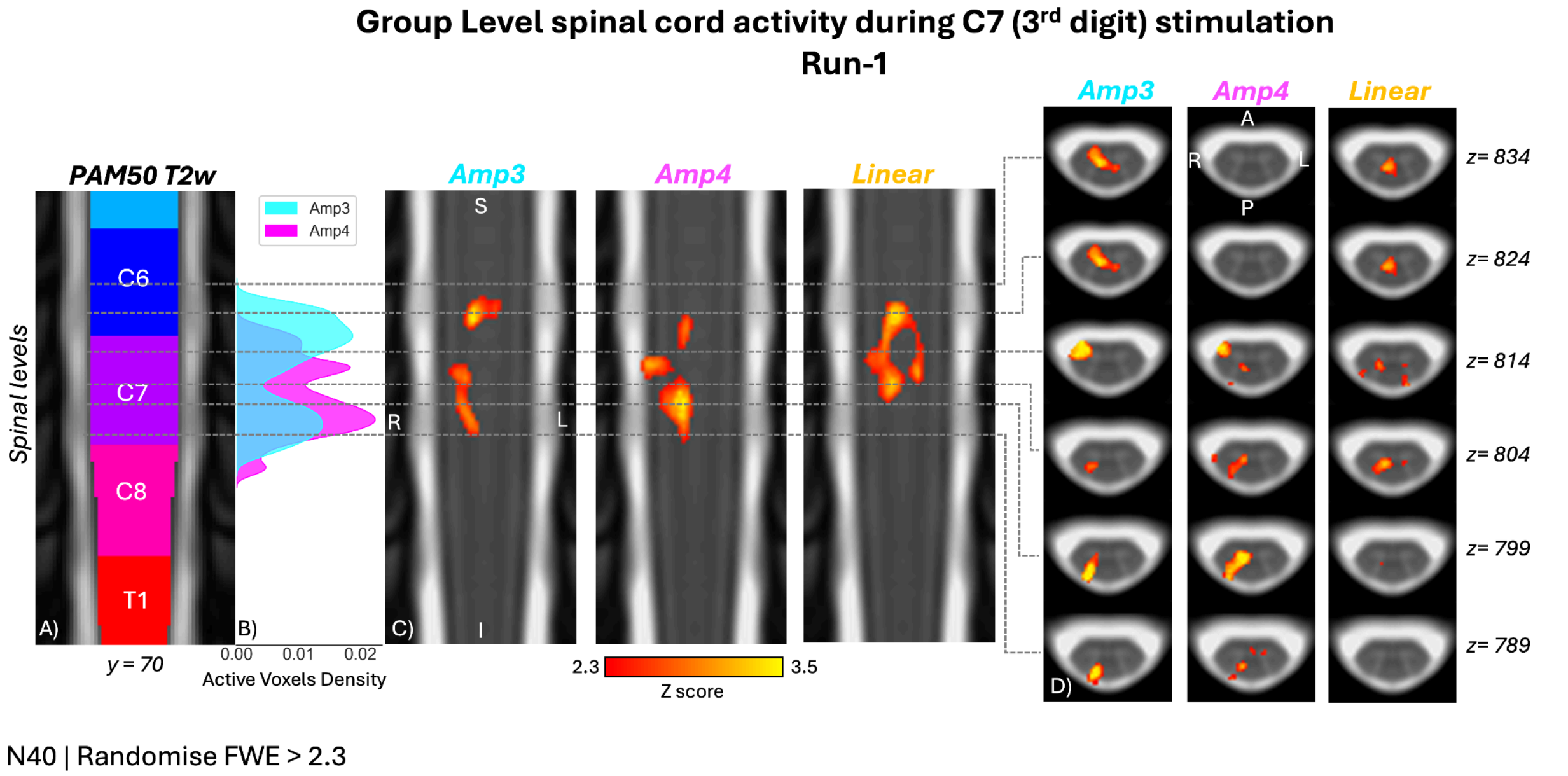

Figure S9. ***Run-1 group-level activity maps during electrocutaneous stimulation of the 3rd digit of the right hand (N = 40, Linear) for run-1. A)*** *Coronal view (y=70, PAM50) with spinal segment levels.* ***B)*** *Active voxel density.* ***C)*** *Coronal slices with activity overlay.* ***D)*** *Axial views of representative clusters. Maps: Randomise, Z score > 2.3, and cluster corrected for family-wise error (FWE) at p < 0.050.*

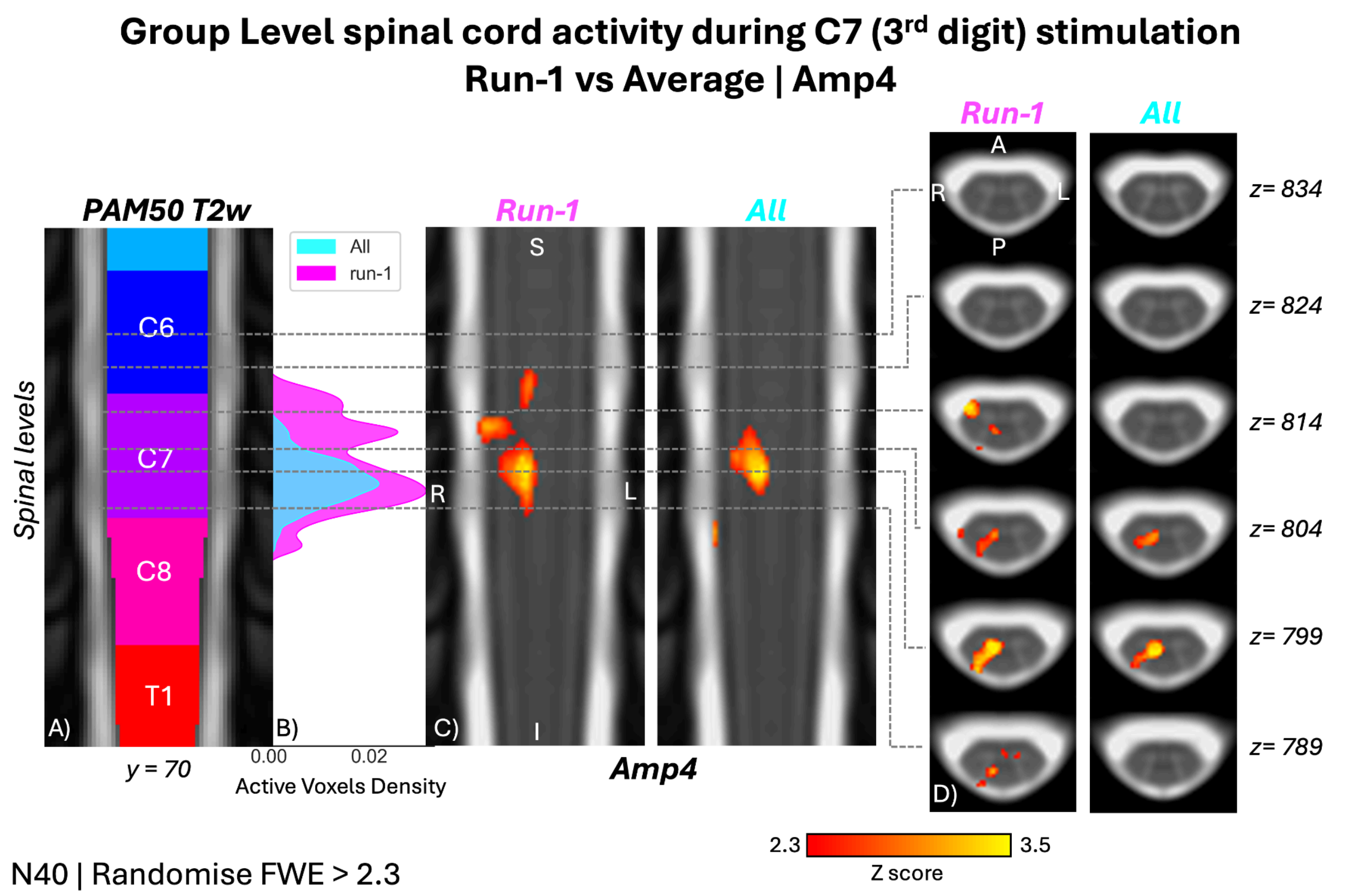

Figure S10. ***Run-1 and average of runs group-level activity maps during electrocutaneous stimulation of the 3rd digit of the right hand (N = 40, Linear) comparing run-1 only vs. average of 3 runs. A)*** *Coronal view (y=70, PAM50) with spinal segment levels.* ***B)*** *Active voxel density.* ***C)*** *Coronal slices with activity overlay.* ***D)*** *Axial views of representative clusters. Maps: Randomise, Z score > 2.3, and cluster corrected for family-wise error (FWE) at p < 0.050.*

12.1. How many participants are needed for a spinal cord fMRI study?

Figure S11 illustrates the voxelwise detection frequency across 1,000 Monte Carlo iterations for Amp4 (averaged across three runs) as a function of sample size (Randomise with Z > 2.3 and FWE cluster correction p < 0.050). As the sample size increases, the cluster emerging at the C7 spinal segment level becomes more stable. Detection frequency surpasses 50% at approximately N ≈ 28, and larger cohorts yield more focal and reliable activity patterns that converge toward the full-cohort (N = 40) group level result.

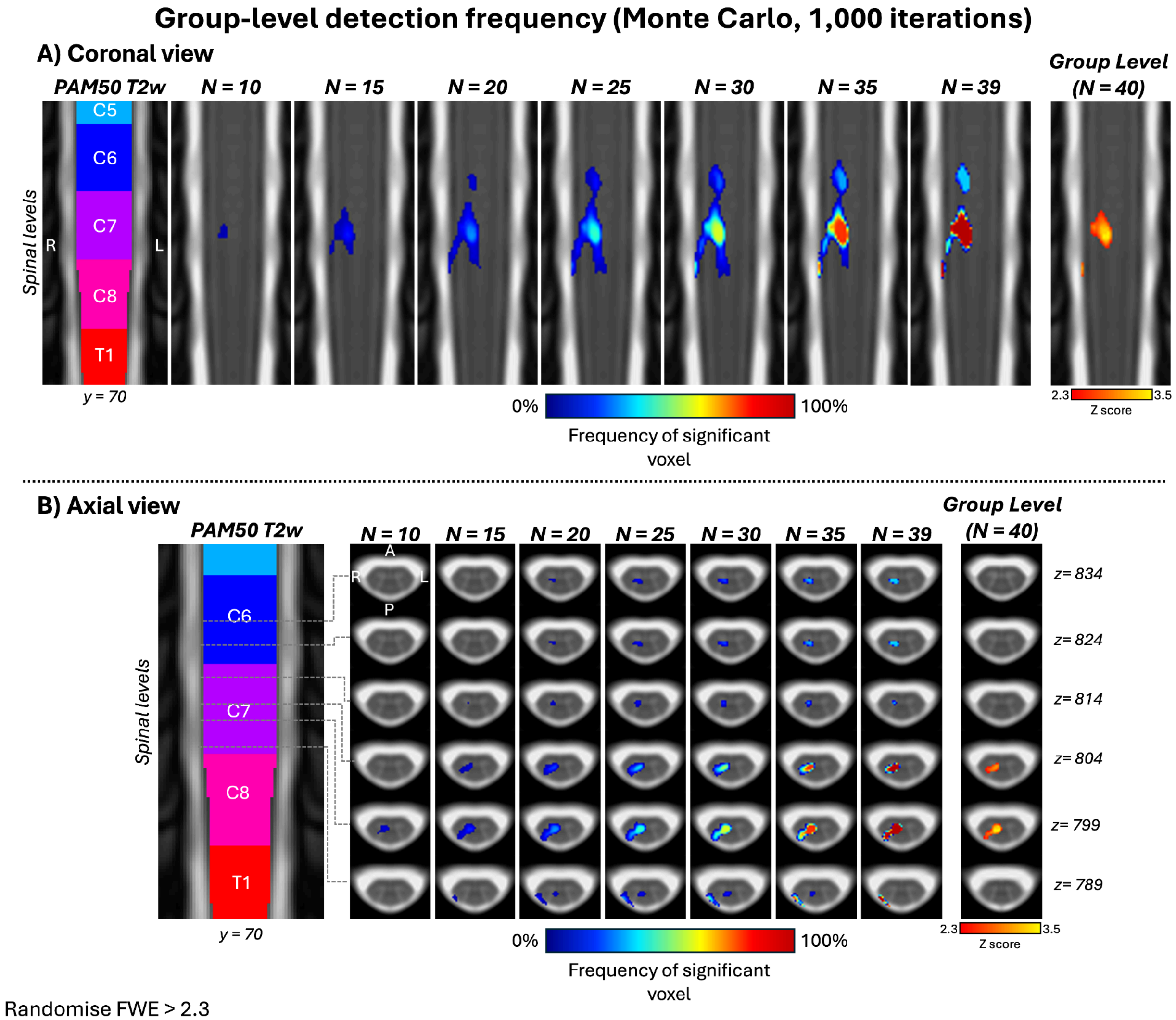

Figure S11. ***Group level detection frequency map across sample sizes.*** ***A)*** *Coronal and* ***B)*** *axial views showing voxelwise detection frequency maps derived from group-level analyses using Randomise with Z > 2.3 and family-wise error (FWE) cluster correction. activity corresponds to the Amp4 stimulation, averaged across three runs. For each sample size (N = 10, 15, 20, 25, 30, 35, 39), subsamples were drawn without replacement, and 1,000 Monte-Carlo iterations were performed to compute the percentage of iterations in which each voxel was significant. The rightmost column shows the corresponding full group-level map (N = 40) for comparison.*
